# Genome-wide association study on metabolite accumulation in a wild barley NAM population reveals natural variation in sugar metabolism

**DOI:** 10.1371/journal.pone.0246510

**Published:** 2021-02-16

**Authors:** Mathias Ruben Gemmer, Chris Richter, Thomas Schmutzer, Manish L. Raorane, Björn Junker, Klaus Pillen, Andreas Maurer

**Affiliations:** 1 Institute of Agricultural and Nutritional Sciences, Chair of Plant Breeding, Martin Luther University Halle-Wittenberg, Halle, Germany; 2 Institute of Pharmacy, Martin Luther University Halle-Wittenberg, Halle, Germany; Murdoch University, AUSTRALIA

## Abstract

Metabolites play a key role in plants as they are routing plant developmental processes and are involved in biotic and abiotic stress responses. Their analysis can offer important information on the underlying processes. Regarding plant breeding, metabolite concentrations can be used as biomarkers instead of or in addition to genetic markers to predict important phenotypic traits (metabolic prediction). In this study, we applied a genome-wide association study (GWAS) in a wild barley nested association mapping (NAM) population to identify metabolic quantitative trait loci (mQTL). A set of approximately 130 metabolites, measured at early and late sampling dates, was analysed. For four metabolites from the early and six metabolites from the late sampling date significant mQTL (grouped as 19 mQTL for the early and 25 mQTL for the late sampling date) were found. Interestingly, all of those metabolites could be classified as sugars. Sugars are known to be involved in signalling, plant growth and plant development. Sugar-related genes, encoding mainly sugar transporters, have been identified as candidate genes for most of the mQTL. Moreover, several of them co-localized with known flowering time genes like *Ppd-H1*, *HvELF3*, *Vrn-H1*, *Vrn-H2 and Vrn-H3*, hinting on the known role of sugars in flowering. Furthermore, numerous disease resistance-related genes were detected, pointing to the signalling function of sugars in plant resistance. An mQTL on chromosome 1H in the region of 13 Mbp to 20 Mbp stood out, that alone explained up to 65% of the phenotypic variation of a single metabolite. Analysis of family-specific effects within the diverse NAM population showed the available natural genetic variation regarding sugar metabolites due to different wild alleles. The study represents a step towards a better understanding of the genetic components of metabolite accumulation, especially sugars, thereby linking them to biological functions in barley.

## Introduction

The importance of metabolites in different areas of life is essential. Metabolomics (parallel to the terms genomics, transcriptomics and proteomics) means the investigation of the metabolome of a living organism and includes the identification and quantification of metabolites, as well as their interactions [1]. Modifications in genes and proteins change the metabolite profile of an organism [2]. Application of metabolomics is wide: from pharmacology over human disease diagnosis to plants [2]. They are used as biomarkers in medicine, for example, to detect lung cancer where certain metabolites show significant differences between healthy and ill individuals [3]. Like for other *omics*, high throughput methods for metabolite screening are available, for instance, a combination of gas chromatography and mass spectrometry (GC-MS) [4]. Estimates for the total number of metabolites in plant kingdom vary from 200,000 to 1,000,000 [5]. In the field of plant breeding, metabolites play an increasingly important role as predictors of phenotype expression in several crop plants, where metabolites are used instead of or in addition to classical genetic markers like SNPs. Different examples are the prediction of complex agronomic traits like yield, heading date and plant height in rice, maize, potato and barley [6–10]. Furthermore, they are involved in abiotic and biotic stress response [11, 12]. For instance, the expression of threhalose-6-phosphate synthase 1 increases drought tolerance in potatoes [13]. Proline plays a key role regarding drought and salt tolerance in different plants [14, 15], phenylpropanoid-polyamine in defence against insect herbivores in *Nicotiana attenuata* and flavonoids for UV light protection [16]. The metabolites built through those stress responses are classified as secondary metabolites, whereas primary metabolites are responsible for plant development. Primary metabolites are controlled by several loci with small effects. In contrast, secondary metabolites underlie the control of a few loci with large effects [17–21]. The increasing investigation of metabolites in plant breeding requires a deeper understanding of the genetic control and the involved genes in metabolite accumulation. Therefore, detecting metabolic quantitative trait loci (mQTL) and candidate genes (CGs), which control the accumulation of specific metabolites, is of great interest [21]. Besides in model plants like *Arabidopsis* genome-wide association studies (GWAS) on metabolite accumulation were successfully applied in the important crop plants rice, wheat and maize with metabolite data from leaves [20, 22, 23] or grains [24]. All studies report a complex genetic architecture of the metabolome, highly influenced by environmental effects [18].

With an acreage of 48.1 m ha in 2017/18, barley (*Hordeum vulgare* L.) is the fourth most important crop worldwide after wheat, maize and rice [25]. Previous studies in barley aiming to find mQTL were focused on stress responses. Drought-adapted genotypes reduced the carbon metabolism in the flag leaf significantly stronger than non-adapted lines [12]. Another study observed changes in ferulic and sinapic acid derivatives as well as acylated glycosides of flavones under drought stress [11]. In both studies, mQTL for different antioxidant metabolites were found. Another study linked the fusarium head blight (FHB) resistance in barley to metabolites belonging mainly to the chemical groups of phenylpropanoids, hydroxycinnamic acid amides, flavonoids, fatty acids, terpenoids and alkaloids [26].

Among metabolites, sugars play a key role in signalling, plant growth and plant development [27–29]. In barley, sugar-related genes are associated with tillering and plant height [30]. Several experiments showed the influence of varying sugar levels on flowering and senescence [31]. Moreover, the role of sugars in defence mechanisms of plants is reported by numerous studies (reviewed in Moghaddam and Van den Ende [32]).

In the present mQTL study, applied in the large barley nested association mapping (NAM) population HEB-25 [33], mQTL for sugars and sugar-like metabolites were detected. Our results promise a better understanding of the interactions of metabolites and phenotypes, as well as the causative genes in barley.

## Materials and methods

### Plant material

The NAM population HEB-25 was generated by crossing and subsequent backcrossing of 25 wild barley accessions (24 *Hordeum vulgare* ssp. *spontaneum* and one *Hordeum vulgare* ssp. *agriocrithon*) with the German elite spring barley cultivar Barke (*Hordeum vulgare* ssp. *vulgare*). The resulting BC_1_S_3_ generation comprises 1,420 individual lines (whereof 1,307 were used in this study) subdivided into 25 families (for a detailed description see Maurer *et al*. [33]).

### Genotypic evaluation

DNA of pooled BC_1_S_3:8_ plants of each line was extracted according to the manufacturer’s protocol, using the BioSprint 96 DNA Plant Kit and a BioSprint work station (Qiagen, Hilden, Germany), and finally dissolved in distilled water at approximately 50 ng/μl for genotyping with the recently developed barley Infinium iSelect 50K chip [34] at TraitGenetics, Gatersleben, Germany. SNP markers that did not meet the quality criteria (polymorphic in at least one HEB family, < 10% failure rate, < 12.5% heterozygous calls as 6.25% is the expectancy in BC_1_S_3_) were removed from the data set. Altogether, 33,005 SNPs met the quality criteria and were analysed in this study. Based on the Barke reference genotype, the wild barley allele can be specified in each segregating family. To setup, the quantitative identity-by-state (IBS) matrix the state of the homozygous Barke allele was coded as 0, while HEB lines that showed a homozygous wild barley genotype were assigned a value of 2. Consequently, heterozygous HEB lines were assigned a value of 1. If an SNP was monomorphic in one HEB family but polymorphic in a second family, lines of the first HEB family were assigned a genotype value of 0, since their state is not different from the Barke allele. Missing genotype calls (0.84%) were estimated by applying the mean imputation (MNI) approach [35]. The genotype matrix is available at e!DAL [36, 37]. The markers are uniformly distributed over the whole genome with few gaps and decreasing density in the telomere regions [10].

### Field trial

In 2017, a field trial with HEB-25 was conducted at the Kühnfeld experimental station of the University of Halle (51°29’45.72"N; 11°59’36.62"E) to gather metabolite data. The trial was sown at the end of march (27^th^/28^th^). Plots consisted of two rows of 50 seeds each with a row length of 1.40 m and a spacing of 0.20 m between rows and 0.50 m between plots. They were completely randomized in a rectangular shape consisting of 18 rows and 82 columns, resulting in a total number of 1,476 plots, surrounded by cultivar Marthe to reduce border effects. Seventeen control genotypes (with 3–8 repeats each) were distributed randomly across the field. Pest control and fertilisation followed local practice.

The studies were conducted on land owned by the authors’ institutions. The research conducted complied with all institutional and national guidelines.

### Metabolic evaluation

Sampling took place on 22 May 2017 under a clear sky between nine and ten o’clock. This date represented the developmental stage BBCH 30–31 (beginning of shooting [38]) for the majority of plants. A 2 cm tissue sample from the middle region of the last fully developed leaf of each HEB line was sampled. The leaf was cut approximately 1 cm from the stem and was put in an Eppendorf tube. The protruding leaf tip was cut off; this resulted in a leaf section of the fully differentiated middle part of the blade as the leaf sample for our analysis. The Eppendorf tube was closed and put instantly in liquid nitrogen to stop metabolic processes. All plots were sampled within one hour under constant weather conditions. In total, 29 people were involved to meet this schedule. Sampling was repeated under the same circumstances (constantly clear sky, equal time of day, equal sampling methods) on 22 June 2017. The plants were more heterogeneous at this time, representing developmental stages BBCH 59–69 (end of ear emergence to end of flowering). The purpose of the second sampling was not simply to repeat the first. Rather, the intention was to find out how the metabolites differ depending on the developmental stage of sampling and what role genetics play.

The frozen leaf samples were pulverised using a Retsch-ball mill (MM 400, Retsch, Germany) for 2 minutes at 20 Hz. The homogenised leaf samples were then resuspended in 700 μl methanol:chloroform:water solution (3:2:4) containing 8 μg/ml ^13^C-sorbitol as an internal quantitative standard. The mixture was shaken for 20 min at room temperature and at 500 rpm. The mixture was then centrifuged for 11,000 X g for 5 minutes at 4°C. After the extraction, 10 μl of the supernatant was dried in a vacuum concentrator without heating for 45 minutes. Online derivatization was performed using the Multi-Purpose Sampler (MPS, Gerstel, Germany) by adding 30 μl Methoxamine hydrochloride (20 mg/ml in Pyridine) to the samples and shaken for 30 min at 45°C. Furthermore, 45 μl N,O-Bis(Trimethylsilyl)trifluoroacetamide and 5 μl Alkane-Standard (C10-C28; 6 mg/ml) were added and the samples were shaken again for 120 min at 45°C. As quality controls for the measurement procedure, leaf samples from 10 randomly chosen Barke reference plants were extracted and pooled together. These standards had the same chemical composition all the time and were used for intra-batch and inter-batch correction of the data analysis. All the samples along with 20% of quality controls were analysed with GC-MS (GC-qTOF system -7890B/7200, Agilent, Santa Clara, USA). One μl of the derivatized samples were injected at 250°C in a splitless mode with a helium gas flow set to 1 ml min^-1^. Chromatography was performed with a 30-m Zebron Capillary GC-Column (ZB-Semi Volatiles, 30 m, 0.25 mm, 0.25 μm). The Helium flow was constant at 1 ml/min. The temperature program was set to 60°C followed by a linear ramp of 10°C/min to 320°C and holding at this temperature for 3 minutes. Throughout the run, the transfer line, source and the quadrupole were set to 290°C, 230°C and 150°C respectively. The raw data was processed by MassHunter Qualitative Analysis software (Agilent, B.07.00) and MassHunter Quantitative Analysis software for QTOF (Agilent, B.08.00). The mass spectra library NIST 14 (National Institute of Standards and Technology) and standard compounds were used for identification and confirmation of the chromatographic peaks. Peak areas were normalized with the internal standard, quality controls and fresh weight.

This resulted in data for 1,307 lines with 158 metabolites (alkanes, amino acids, organic acids, sugars and unknowns). Metabolites, which were under the limit of quantification or saturated, or with > 10% missing values were removed from the data set so that 123 metabolites were used for GWAS (S1 Table). Samples from the 2^nd^ sample date resulted in data for 1,229 lines with 159 metabolites (one additional unknown metabolite). After data cleaning 118 metabolites remained for the subsequent analyses (S2 Table). Remaining missing values were replaced with the minimum value of the respective metabolite.

### Statistical analyses

All statistical analyses were performed with SAS 9.4 [39] and R [40]. Pearson’s correlation coefficients were calculated with R software with the corrgram package [41]. The box-cox power transformation [42] was applied to metabolic data using SAS PROC TRANSREG with λ ranging from -3 to 3 by steps of 0.25. The genomic heritabilities of metabolites (also called SNP-based heritabilities, [43]) were estimated with the R package sommer [44] as $h_{SNP}^{2}=\frac{\sigma_{A}^{2}+\;\sigma_{D}^{2}+\;\sigma_{I}^{2}}{\sigma_{A}^{2}+\;\sigma_{D}^{2}\;+\;\sigma_{I}^{2}+\;\sigma_{R}^{2}}$, where $\sigma_{A}^{2},\;\sigma_{D}^{2},\;\sigma_{I}^{2}$ and $\sigma_{R}^{2}$ represent the additive, dominance, epistatic and residual variance components, respectively. Additionally, repeatability of metabolites was calculated for the subset of 17 genotypes (elite cultivars, control lines) where multiple metabolite measurements were available as $rep=\frac{V_{G}}{V_{G}+\;\frac{V_{R}}{r}}$, where *V*_*G*_ and *V*_*R*_ represent the genetic and residual variance components, respectively, while *r* represents the number of replications per genotype. Descriptive statistics for metabolites were calculated with R package psych [45]. All figures were created with R using the package ggplot2 [46]. Fig 2 was created with the tool InteractiveVenn [47].

### Genome-wide association study (GWAS)

We used a multiple linear regression model with SNP markers being included as main effects using the quantitative IBS genotype matrix scores, to conduct genome-wide association mapping for each Box-Cox transformed metabolite. The analysis was carried out by means of model selection with SAS PROC HPREG. This procedure can select the best model based on a set of predefined possible factors. In our case, all 33,005 SNPs were initially defined as possible factors. Significant SNPs were then determined by stepwise forward-backward regression. SNPs were allowed to enter or leave the model at each step based on the p-value (< 0.001) calculated for the marginal F-test of that term. SNPs included in the final model are hereafter referred to as significant SNPs. An SNP’s effect estimate can be interpreted as the allele substitution effect and represents the regression coefficient of the respective SNP in the final model. Note that all significant SNPs’ effect estimates are modelled at the same time in the final model. Five-fold cross-validation was run to increase the robustness of the results. For this, the lines were divided into 5 folds with each fold consisting of 20% randomly-chosen HEB lines per family. Each possible combination of 4 different folds was then used as the training set to define significant markers and to estimate their effects based on the above-mentioned model selection procedure, while the remaining fold was used as the validation set. The metabolite data of the validation set lines was predicted based on marker effects estimated in the training set. Prediction ability (r^2^) was then calculated as the squared Pearson product-moment correlation between the observed and predicted metabolite data of the validation set. In total, this procedure was repeated 20 times with different random creation of folds, ergo in total 100 cross-validation runs were performed. To define mQTL regions, we calculated an SNP marker’s detection rate (DR) as the number of times, out of 100 cross-validation runs, it was included in the final model. We defined an mQTL as robust if DR ≥ 25. This threshold was set after a permutation test based on three shuffled genotype-metabolite matrices used for the above-mentioned cross-validation procedures (≙ 300 cross-validation runs). Based on the results obtained from that we observed a detection rate of 25 as the 99.99% percentile (that means 99.99% of markers have less detections). If the observed detection rate from the original data was exceeding this threshold, we declared the presence of a significant marker-metabolite association. For the calculation of the explained phenotypic variance of a single mQTL, all SNPs exceeding the DR in the respective mQTL region were fitted in a multiple linear regression model to explain the metabolite phenotype in the whole dataset. To estimate a family-specific mQTL effect we applied the cumulation method as presented in Maurer et al. [48]. This procedure was conducted within each of the 100 cross-validation runs and the mean of them was taken as the final family-specific mQTL effect estimate.

## Results and discussion

### Metabolic data and GWAS performance

**Fig 1:**
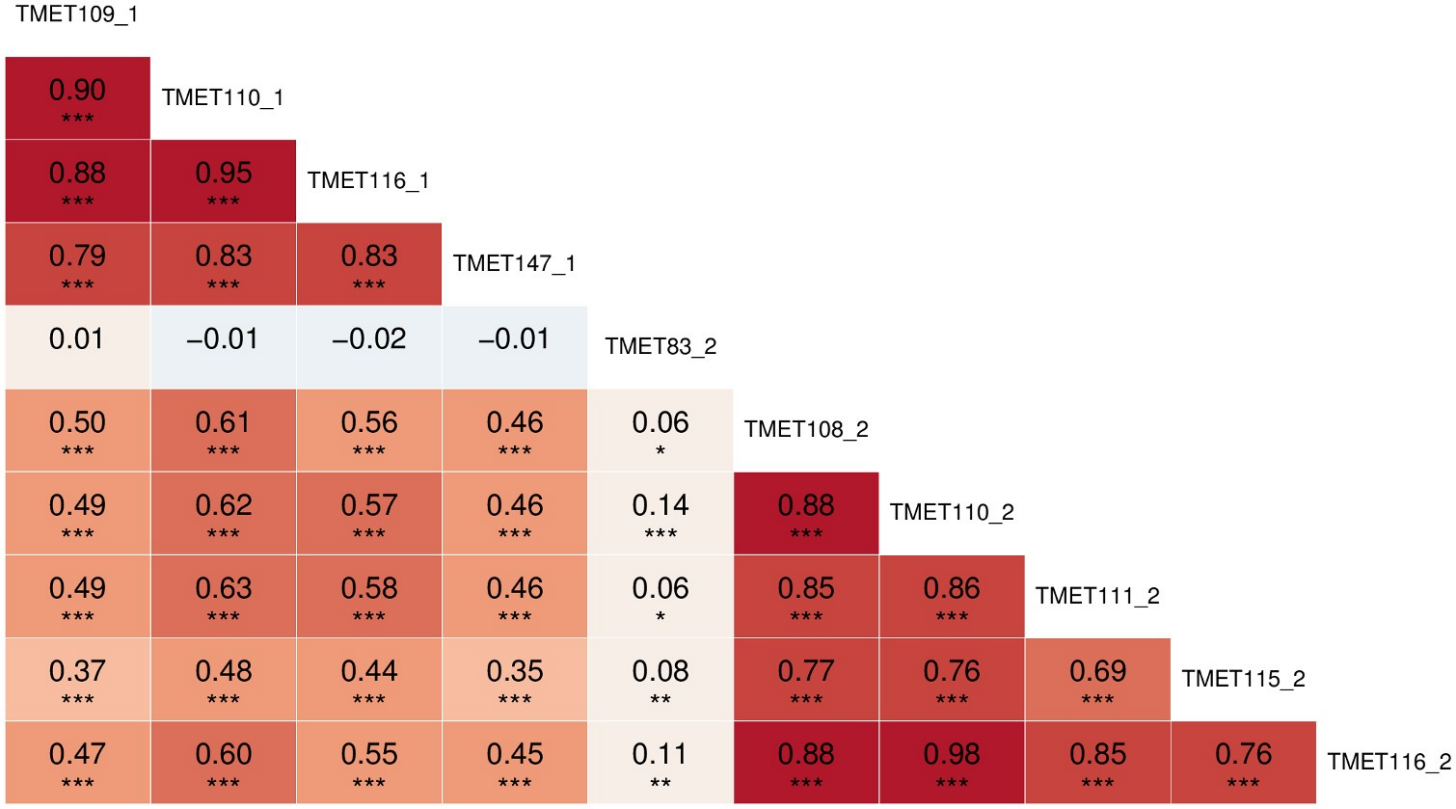
Pearson’s correlation coefficients of metabolites investigated in this study. Significant correlation coefficients are indicated with * p < 0.05, ** p < 0.01 and *** p < 0.0001. The colour intensity indicates the strength of the correlation. Red indicates positive, blue indicates negative correlation. Metabolites from the first and second sampling date are highly correlated among themselves, with the exception of TMET83_2. The correlations between the first and second sample date are also medium positive and highly significant.

The analysed metabolite set includes amino acids, fatty acids, organic acids and sugars. For most of the unknown metabolites, at least the substance group (mostly sugar) is known. S1 and S2 Tables show a detailed list of all determined metabolites and their substance grouping. Each metabolite’s variation is illustrated in S1 and S2 Figs. The concept of estimating SNP-based heritability [43], also called genomic heritability, was already applied in Gemmer et al. 2020 [10] to the metabolite data resulting in values of up to 0.50 (S3 and S4 Tables). Repeatabilities of metabolite measurements showed high variation across metabolites (0.00–0.87) with mean values of 0.26 and 0.28 (S3 and S4 Tables), hinting on limited data quality for several metabolites that may affect QTL detection. In this context it is noticeable, that the metabolites with sufficiently high prediction abilities (mean r^2^ > 0.2) in GWAS (TMET109_1, TMET110_1, TMET116_1, TMET147_1, TMET83_2, TMET108_2, TMET110_2, TMET111_2, TMET115_2, TMET116_2, with _1 and _2 indicating the first or second sampling date; Table 1), showed above-average SNP-based heritabilities as well as repeatabilities (with exception of TMET115_2, repeatability = 0.22, S4 Table). There were clearly positive correlations between the genomic heritability of metabolites and their estimated mean r^2^ values (prediction ability) in GWAS at both sampling dates (S3 and S4 Tables, S3 and S4 Figs). All of these metabolites are unknowns, with the exception of TMET83_2, which represents threonic acid (substance group sugar acid). However, substance groups of the remaining metabolites are known. TMET109_1, TMET116 (both sampling dates), TMET147_1, TMET108_2, and TMET111_2 are classified as sugar-like metabolites, while TMET115_2 is a disaccharide. Only TMET110 (both sampling dates) is completely unknown. The correlation pattern among metabolites mostly reflects the substance grouping of them (S5 and S6 Tables, S5 and S6 Figs). For instance, seven sugars of the first sampling date clustered together, including the four ones for which mQTL were obtained. Metabolites from the first and second sampling date correlated just slightly, with the exception of one hotspot. Interestingly, this hotspot comprises all those metabolites from the first sampling date and five out of six metabolites from the second sampling date for which mQTL were obtained (S7 Table and S7 Fig). Fig 1 illustrates this correlation, including the metabolite TMET83_2, the only metabolite in the mQTL study which was not correlated with the others. Due to the strong correlation of TMET110 with the other unknown sugar-like metabolites, this metabolite can with a high possibility be classified as sugar-like, too.

**Table 1:** Summary of GWAS results.

Metabolite^a^	r^2^ GWAS^b^	Number of significant SNPs^c^	r^2^ of major mQTL-1H^d^
TMET109_1	0.24	17	0.38
TMET110_1	0.51	15	0.65
TMET116_1	0.40	15	0.53
TMET147_1	0.21	14	0.36
TMET83_2	0.43	10	-
TMET108_2	0.37	12	0.32
TMET110_2	0.27	23	0.64
TMET111_2	0.35	15	0.15
TMET115_2	0.20	14	0.36
TMET116_2	0.26	25	0.53

^a^ Metabolites, see S1 and S2 Tables; _1 and _2 indicate 1st and 2nd sampling date

^b^ Mean cross-validated r^2^ value (prediction ability) of the metabolite in GWAS

^c^ Number of significant SNPs in GWAS, DR ≥ 25

^d^ r^2^ value (unvalidated) of the major mQTL-1H estimated in GWAS.

### Metabolic QTL (mQTL) analysis

**Fig 2:**
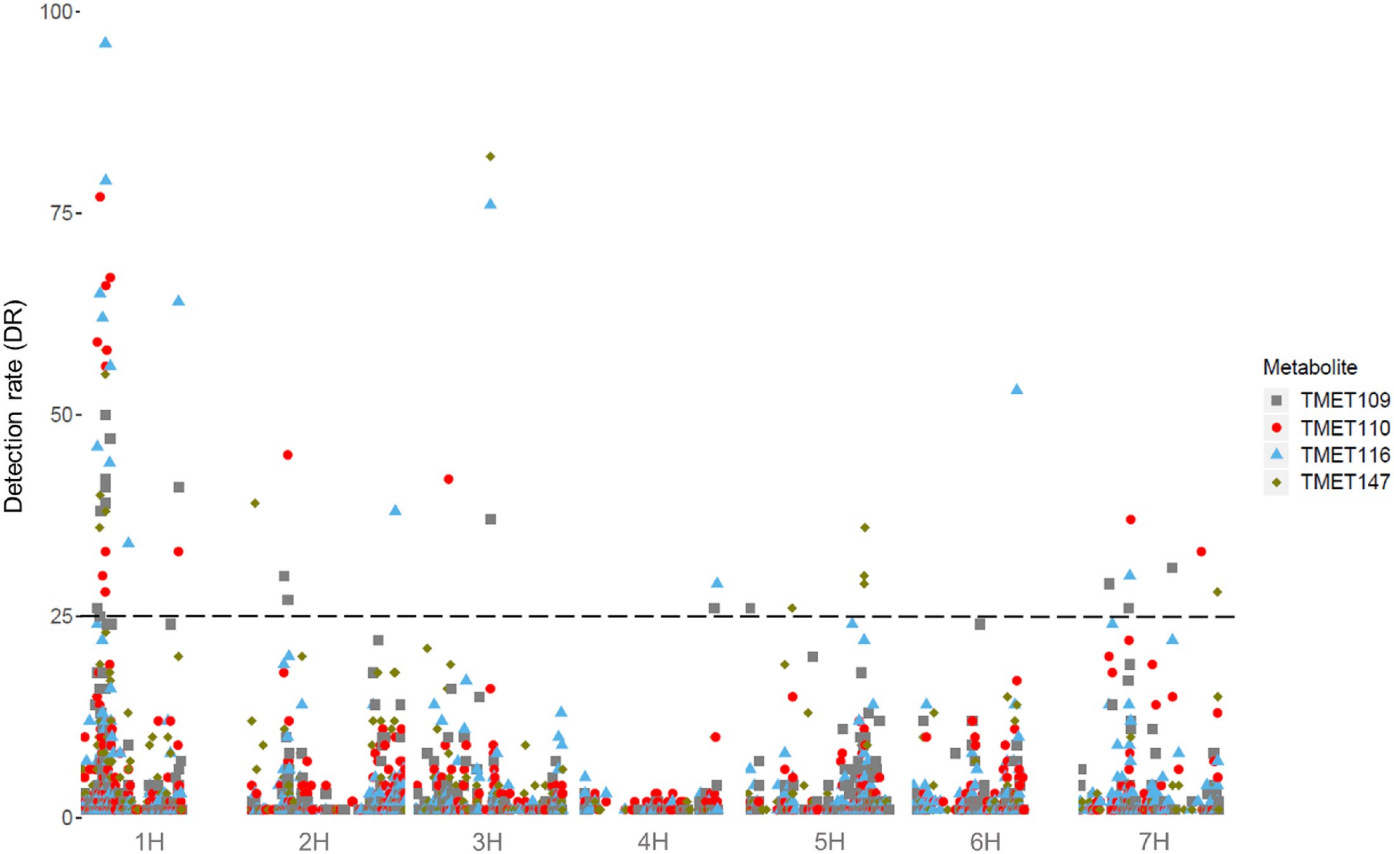
Manhattan plot of all metabolites of the first sampling date. All metabolites showed significant marker-metabolite associations on chromosome 1H. The different point shapes and point colours differentiate the metabolites and the associated SNPs. The x-axis shows the chromosomes with SNP ordering based on the Morex Reference Sequence 1.0 [49], the detection rate (DR) is given on the y-axis. The dashed line indicates the threshold of DR > 25, which was used as significance threshold.

**Fig 3:**
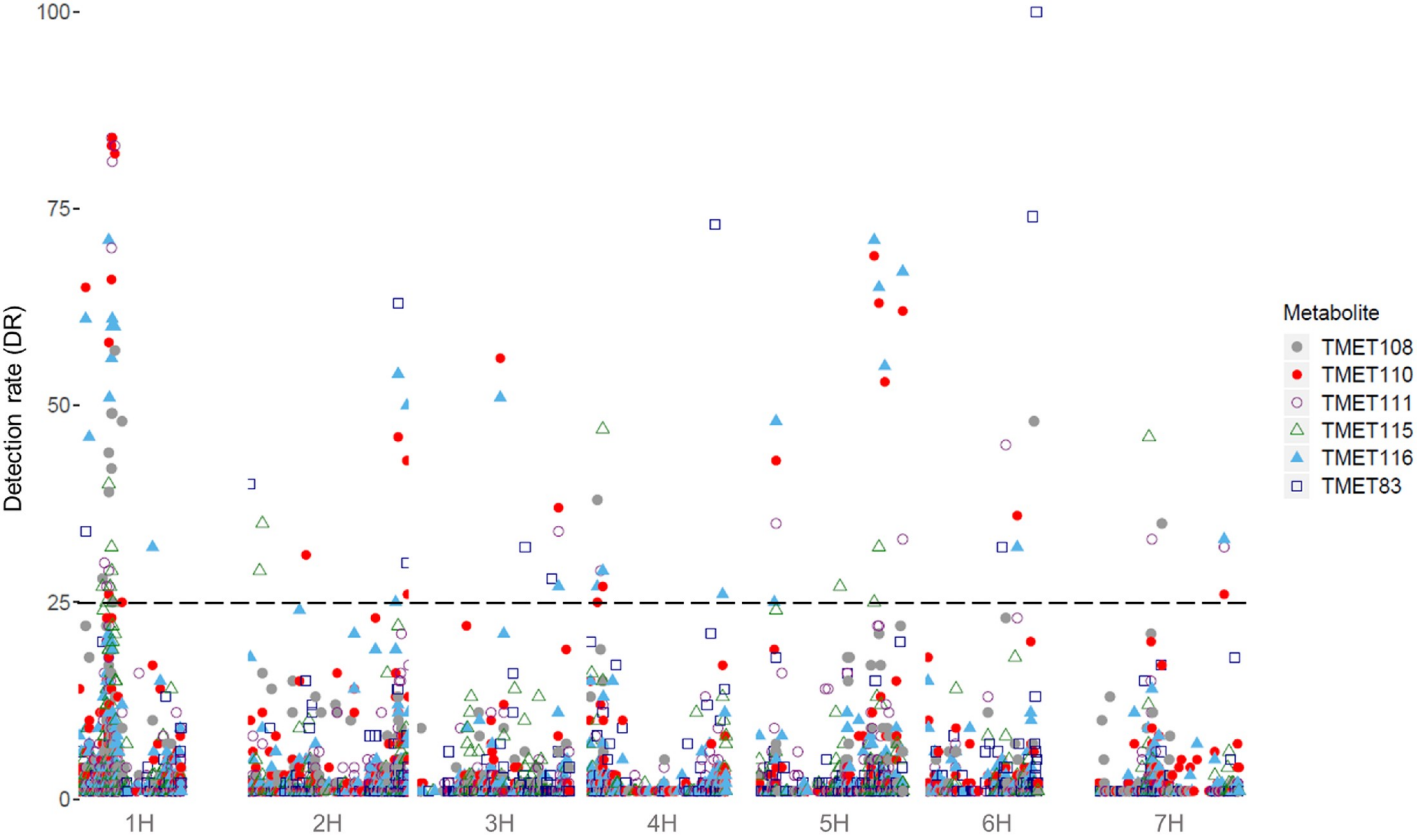
Manhattan plot of all metabolites of the second sampling date. All metabolites showed significant marker-metabolite associations on chromosome 1H. The different point shapes and point colours differentiate the metabolites and the associated SNPs. The x-axis shows the chromosomes with SNP ordering based on the Morex Reference Sequence 1.0 [49], the detection rate (DR) is given on the y-axis. The dashed line indicates the threshold of DR > 25, which was used as significance threshold.

**Fig 4:**
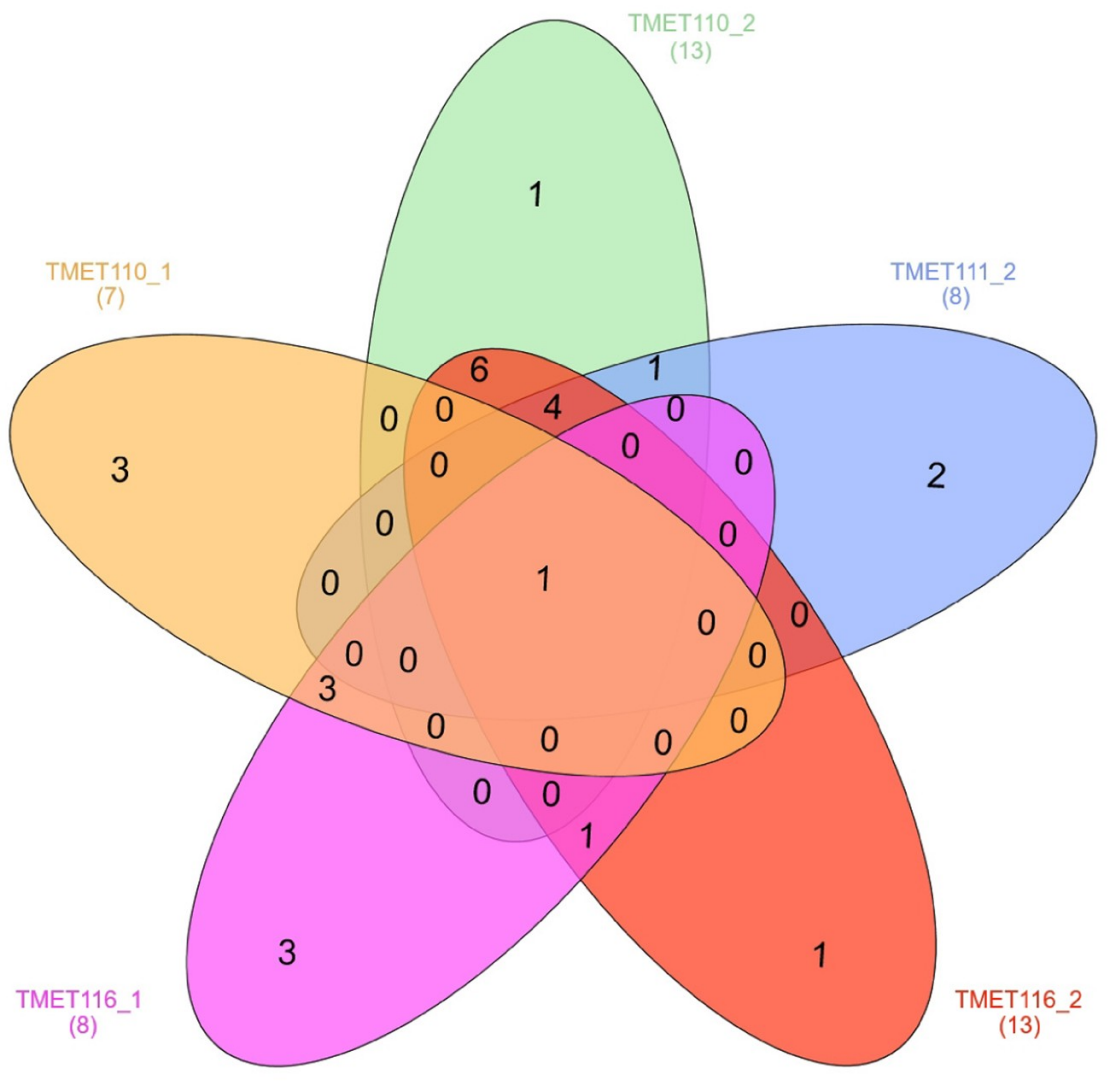
Number of shared mQTL between five correlated metabolites. All metabolites share one mQTL, the major mQTL on chromosome 1H.

Only eight metabolites with prediction abilities (r^2^) > 0.2 were further screened for mQTL, which were defined if a SNP reached a detection rate (DR) of ≥ 25 in 100 cross-validation runs. Under these circumstances, mQTL for four metabolites from the first and six metabolites from the second sampling date were detected, with between ten and 25 associated SNPs for each metabolite (Table 1, Figs 2 and 3). Those could be grouped into 19 mQTL for the first sampling date and 25 mQTL for the second sampling date (S8 and S9 Tables). Two metabolites with significant marker-metabolite associations were found at both sampling dates (TMET110 and TMET116). This slight overlap confirms our expectation, that at different developmental stages different metabolites are produced. The maximum r^2^ value was 0.51 for metabolite TMET110_1. Most significant SNPs were found for TMET116_2 (25). S10 Table shows the complete results of GWAS (DRs of all significant markers for all metabolites, estimated effects of marker-metabolite associations for all metabolites). Some mQTL were shared between metabolites or sampling dates (Table 2, Fig 4). The results of the ten most interesting mQTL (Table 2) are discussed in detail below.

**Table 2:** List of selected mQTL and sugar-related candidate genes.

Metabolite^a^	mQTL^b^	Position Ref. Seq. 1.0 (bp)^c^	Total number of genes^c^	Selected candidate genes (CGs)^d^
83_2	1H-1_2	4,228,359–6,332,167	65	Serine/threonine-protein kinase
108_2; 110_2; 111_2; 115_2; 116_2	1H-2_2	13,579,164–20,291,169	98	Disease resistance proteins, UDP-galactose transporter 5
109_1; 110_1; 116_1; 147_1	1H-1_1	14,371,313–20,291,169	82	Disease resistance proteins, UDP-galactose transporter 5
108_2	1H-3_2	28,823,904	27	Nucleotide-sugar transporter family protein
116_1	1H-2_1	36,457,092	25	Sugar transporter 1
116_2	1H-4_2	444,533,575	20	Trehalose-6-phosphate phosphatase
83_2	2H-3_2	691,870,056	40	Serine/threonine-protein kinase
115_2	5H-2_2	503,110,566	6	Sugar transporter protein 7
83_2; 108_2	6H-3_2	570,020,515–580,178,325	247	Sugar transporter SWEET
111_2; 115_2	7H-1_2	47,788,650; 52,114,669	30; 22	GDP-mannose transporter 1

^a^ Metabolites, see S1 and S2 Tables; _1 and _2 indicate 1st and 2nd sampling date.

^b^ mQTL name with chromosome positions and consecutive numbering (see S8 and S9 Tables); _1 and _2 indicate 1st and 2nd sampling date.

^c^ Physical position (in base pairs) of the mQTL, derived from Reference Sequence 1.0 [49] and corresponding number of genes present in the given range or within 2,000,000 bp surrounding the indicated single position.

^d^ Arbitrarily selected candidate genes (derived from Barleymap [50]) in the given range or within 2,000,000 bp surrounding the mQTL peak marker of the mQTL. For exact positions, see S8, S9 and S11 Tables.

There was one outstanding mQTL on chromosome 1H (for simplicity referred as mQTL-1H) in the region of approximately 13 Mbp to 20 Mbp (approx. 27–30 cM). At both sampling dates, all metabolites (with r^2^ >0.2 and DR ≥ 25) except TMET83_2 showed significant marker-metabolite associations in this genomic region. High amounts of up to 65% (TMET110) of explained phenotypic variance of the respective metabolites could be attributed to this single mQTL (Table 1, S12 Table). While in case of TMET110 this mQTL seems to be the main responsible mQTL for metabolite accumulation, other metabolites like TMET111_2 with no more than 15% of explained phenotypic variance indicate a more complex genetic control. The detection of further relevant mQTL for TMET111_2, TMET108_2, and TMET115_2 supports this assumption. Interestingly, the explained phenotypic variance of mQTL-1H for TMET116 was the same for both sampling dates, as in the case of TMET110. This hints on a stable genetic impact on these metabolites’ accumulations at both plant developmental stages.

In the corresponding chromosomal region genes coding for resistance proteins as well as for UDP-galactose transporter 5 (HORVU1Hr1G008350) and a Glucose-6-phosphate isomerase (HORVU1Hr1G006860) are located (S8, S9 and S11 Tables). Besides this major mQTL, several further significant marker-metabolite associations in the proximity of disease resistance-related genes were detected (S8 and S9 Tables). This indicates the role of sugar metabolites in the plant defence system, where they are primarily involved in signalling [28, 29, 32]. Sugars activate genes, which recognize pathogen-associated molecular patterns (PAMPs) [51]. In this context, research on sugars and sugar-like compounds as an alternative to chemical plant protection is of great interest [52].

UDP-Galactose transporter 5 belongs to the group of nucleotide-sugar transporters. In general, sugar transporters are responsible for the distribution of sugars in the plant. Short (cell-to-cell) and long-distance transports are distinguished. Besides nutrition, they provide sugars for cells involved in growth and development. Moreover, they are important for signalling [53]. Besides the major mQTL mQTL-1H-1_1 and mQTL-1H-2_2, also mQTL-1H-2_1, mQTL-1H-3_2, mQTL-5H-2_2, mQTL-6H-3_2 and mQTL-7H-1_2 include different sugar transporter CGs, namely: Sugar transporter 1, a nucleotide-sugar transporter family protein, sugar transporter protein 7, sugar transporter SWEET and GDP-mannose transporter 1. The mQTL and CGs correspond to all eight metabolites.

As UDP-galactose transporter 5, also nucleotide-sugar transporter family proteins and GDP-mannose transporter 1 belong to the group of nucleotide-sugar transporters. They are located in the Golgi apparatus and the endoplasmic reticulum of eukaryotic cells [54]. Nucleotide sugars are substrates of glycosyltransferases and are synthesized in the cytoplasm. Glycosyltransferases need them to equip proteins and lipids with sugar rests. Glycosylation reactions mainly take place in the Golgi apparatus. Via these nucleotide-sugar transporters, the sugars are transported to the reaction locus [55]. Sugar transporter 1 and sugar transporter protein 7 are unspecific sugar transporters which transport sugars through the cell membrane. SWEET sugar transporters are responsible for the transport of hexose and sucrose for different purposes like seed filling and nectar secretion [56]. Moreover, the expression of SWEET genes is induced by fungal and bacterial pathogens. This indicates that the sugar efflux function of the transporters is the target of pathogens to ensure their own nutrition [57] and further hints on sugar metabolites’ involvement in pathogen responses.

Glucose-6-phosphate isomerase is an essential enzyme of catabolic glycolysis and anabolic gluconeogenesis that catalyses the reversible isomerization of glucose-6-phosphate and fructose-6-phosphate [58]. This reaction is essential for all living organism to utilize the energy from carbohydrates. Regarding plants, it was identified to be a salt-induced protein in the green algae *Dunaliella salina* [59]. Under salt stress, glucose-6-phosphate isomerase is increased and involved in adaption to high salinity [59].

TMET83_2, threonic acid, is the only known metabolite with sufficient GWAS performance in our mQTL study. It is a sugar acid derived from threose, which forms the basic structure of the essential amino acid threonine. Four mQTL for TMET83_2 harbour serine/threonine-protein kinases as CGs (on 1H, 2H and 3H), while mQTL6H-3_2 harbours serine/threonineprotein phosphatase 2A (HORVU6Hr1G091550). In general, protein kinases in plants phosphorylate proteins and are involved in many different important processes, mainly in signalling regarding to nutrition, pathogen attacks and abiotic stresses. The impact of protein kinases in plant metabolism is highlighted by the estimation that 1–3% of functional genes account for them [60]. Besides TMET83_2, several protein kinase family protein CGs on different chromosomes for several metabolites were found (S8 and S9 Tables). Serine/threonine protein kinases play a key role in apoptosis. The phosphorylation of different substances determines its function in the apoptotic process, triggered by biotic and abiotic stress [61]. The threonic acid levels observed in this study might, therefore, reflect a plant’s reaction to stress.

For mQTL-1H-4_2 (TMET116_2), trehalose 6-phosphate phosphatase is a potential CG. The phosphorylated form of trehalose is an important regulator of plant growth, development and senescence [62]. Although most correlations of sugar-like metabolites and flowering time were weak (S13 Table), several mQTL co-localize with known CGs influencing plant development (S8 and S9 Tables), namely *HvELF3* (1H), *Ppd-H1* (2H), *Vrn-H1* (5H), *Vrn-H2* (4H) and *Vrn-H3* (7H) [33]. In this regard, TMET109_1 is striking as it is negatively correlated (*r* = -0.21, p < 0.0001) with flowering time and shows significant associations with *HvELF3*, *Ppd-H1*, *Vrn-H2*, and *Vrn-H3* (S8 Table), which hints on its involvement in flowering time regulation or reflects developmental differences at the time of sampling. In general, correlations with flowering time were more pronounced at the first sampling date, which is likely due to the higher diversification of flower initiation during this time than at the second sampling date, when most genotypes had already flowered. However, to link metabolites to phenotypes a model which incorporates all metabolites simultaneously is advisable [10].

The fact that mQTL for different metabolites were reliably detected reflects that genetic variation for these metabolites’ accumulations is present in the HEB-25 population. To investigate which of the 25 diverse donor alleles in HEB-25 cause the differences in the respective metabolites, family-specific effects for the detected mQTL were computed (S14 and S15 Tables). In the case of mQTL-1H, which showed the highest impact on all of the metabolites, the estimated effects varied considerably between families. Depending on the family, this mQTL causes effects of different strength or even different directions, with half of the families showing little difference to the cultivated allele. Interestingly, the family-specific effects of the different metabolites correspond to each other, i.e. mQTL-1H has a similar impact on different metabolites, which indicates that those metabolites are closely related and based on the same genetic regulation. This is true for both sampling dates. At the 2^nd^ sampling date, family 06 showed at several mQTL (e.g. mQTL2H-3_2 and mQTL5H-4_2) the most extreme effects as compared to the other families, especially for TMET110 und TMET116. So does family 16 at mQTL3H-3_2. Looking at TMET83, the only non-sugar metabolite, mQTL6H-3_2 causes high effects of the same direction in all families, indicating that all wild alleles exhibit a clear difference to the cultivated allele.

### Conclusions

The different direction of effects and thus the variation in metabolites depending on families shows the influence of different wild barley backgrounds (25 different wild barley accessions) on metabolite profiles. The fact that besides one exception all metabolites for which mQTL were detected are sugars also allows some conclusions. As discussed above, sugars play an important role in disease resistance and plant development. Due to the introgressed wild barley genome in HEB-25, variation was generated in these traits, which is missing in the genetically narrow modern elite cultivars. The driving force for the variation in metabolites in the present study might have been different resistance mechanisms and developmental differences at the time of sampling. For GWAS, sufficient heritability is essential to detect QTLs. Low SNP-based heritabilities and repeatabilities in the majority of metabolites might have been the reason that only a few reliable results were obtained by GWAS.

Because of the large population size we were not able to realize more biological and technical replicates for metabolite determination per genotype, which might have increased overall data quality in our study. To overcome these limitations, an approach for further studies could be to create a subset of the most interesting lines (for example, the most extreme lines in terms of metabolite content). Within this small subset, replicates are possible and a validation of our results can be realized. Moreover, selecting genotypes with similar flowering time might help to avoid a bias caused by different developmental stages at sampling. To sum up, data quality should be improved in future studies to obtain satisfying GWAS results for more metabolites.

Our study underlines the importance of sugars in plant metabolism and their impact on plant development, pathogen defence and signalling. It is the first step of further studies which are necessary to investigate the complex interaction of phenotype, genotype and metabolites in barley.

## Supporting information

S1 Fig Histograms of metabolites (after box-cox transformation) from 1^st^ sampling date. (PDF)

S2 Fig Histograms of metabolites (after box-cox transformation) from 2^nd^ sampling date. (PDF)

S3 Fig Estimated mean r^2^ value of metabolites in GWAS (r^2^ GWAS) plotted against SNP based heritability (h^2^) of metabolites, 1^st^ sampling date. (PDF)

S4 Fig Estimated mean r^2^ value of metabolites in GWAS (r^2^ GWAS) plotted against SNP based heritability (h^2^) of metabolites, 2^nd^ sampling date. (PDF)

S5 Fig Heatmap of correlation pattern among metabolites from 1^st^ sampling date. (PDF)

S6 Fig Heatmap of correlation pattern among metabolites from 2^nd^ sampling date. (PDF)

S7 Fig Heatmap of correlation pattern between metabolites from 1^st^ and 2^nd^ sampling date. (PDF)

S1 Table List of metabolites 1^st^ sampling date. (XLSX)

S2 Table List of metabolites 2^nd^ sampling date. (XLSX)

S3 Table Descriptive statistics for the metabolites from 1^st^ sampling date. (XLSX)

S4 Table Descriptive statistics for the metabolites from 2^nd^ sampling date. (XLSX)

S5 Table Correlation matrix (Pearson’s correlation coefficients) of metabolites from 1^st^ sampling date. (XLSX)

S6 Table Correlation matrix (Pearson’s correlation coefficients) of metabolites from 2^nd^ sampling date. (XLSX)

S7 Table Correlation matrix (Pearson’s correlation coefficients) of metabolites from 1^st^ and 2^nd^ sampling date. (XLSX)

S8 Table Detailed list of mQTL of metabolites from 1^st^ sampling date. (XLSX)

S9 Table Detailed list of mQTL of metabolites from 2^nd^ sampling date. (XLSX)

S10 Table Detection rate and mean effect of each marker for metabolites of both sampling dates. (XLSX)

S11 Table Detected SNP markers for the metabolites of both sampling dates. (XLSX)

S12 Table Explained phenotypic variance (r^2^) of defined mQTL regions for investigated metabolites. (XLSX)

S13 Table Pearson’s correlation coefficients between flowering time and metabolites, both sampling dates. (XLSX)

S14 Table Estimated family-specific effects for detected mQTL, 1st sampling date. (XLSX)

S15 Table Estimated family-specific effects for detected mQTL, 2^nd^ sampling date. (XLSX)

S1 File Raw phenotype data. (XLSX)

S2 File Raw metabolite data. (XLSX)

## Abbreviations

CG: *Candidate gene*
FHB: *Fusarium head blight*
GC-MS: *Gas chromatography-mass spectrometry*
GWAS: *Genome-wide association study*
MNI: *Mean imputation*
mQTL: *Metabolic quantitative trait locus*
NAM: *Nested association mapping*
PAMP: *Pathogen-associated molecular patterns*
SNP: *Single nucleotide polymorphism*
