# Supplementary figures and images for: Genome-wide association study on metabolite accumulation in a wild barley NAM population reveals natural variation in sugar metabolism
Source: PLoS One. 2021 Feb 16;16(2):e0246510. doi: 10.1371/journal.pone.0246510 (PMC7886226; doi:10.1371/journal.pone.0246510)

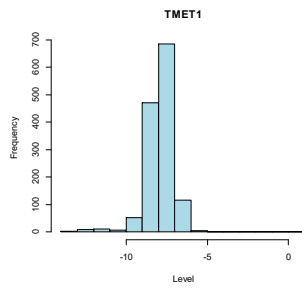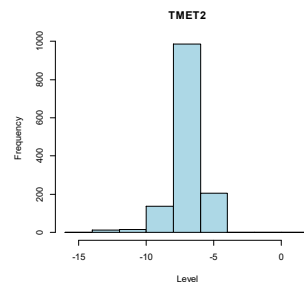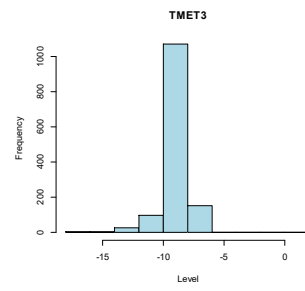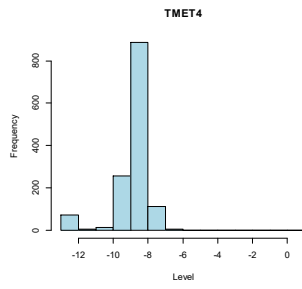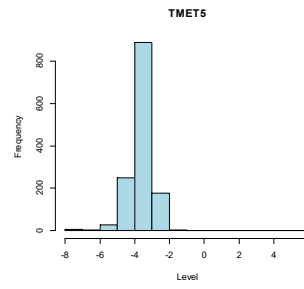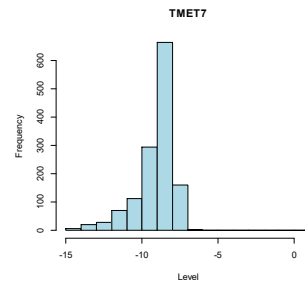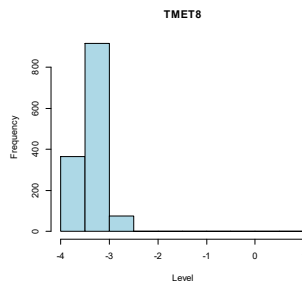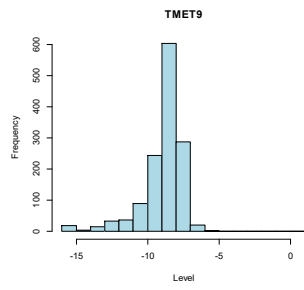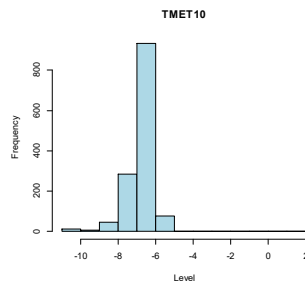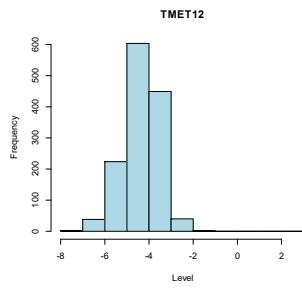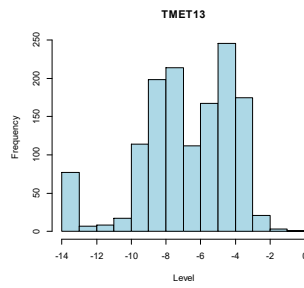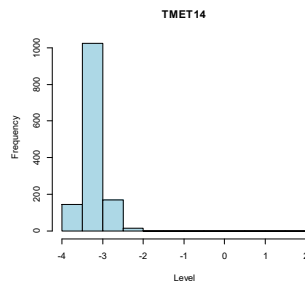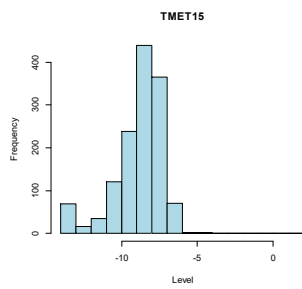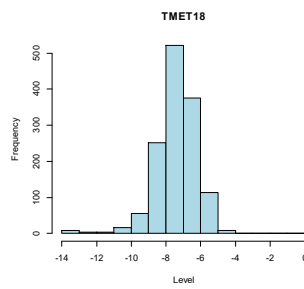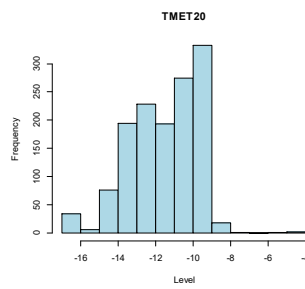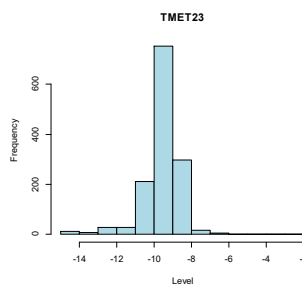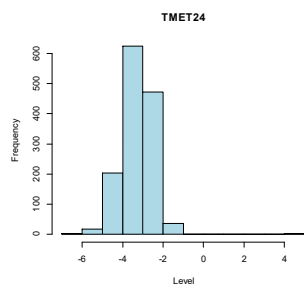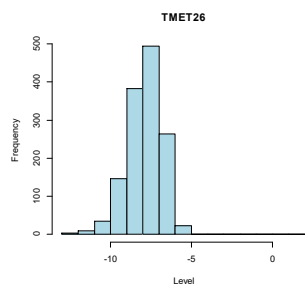

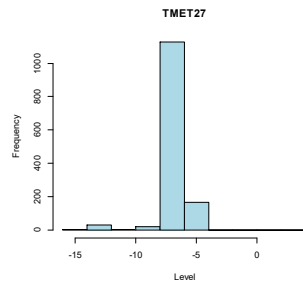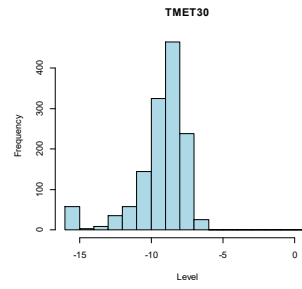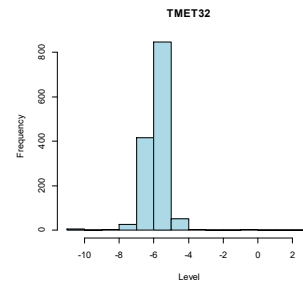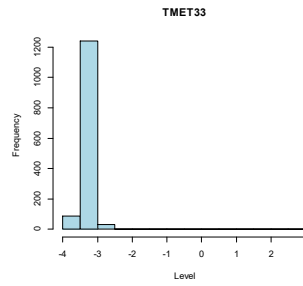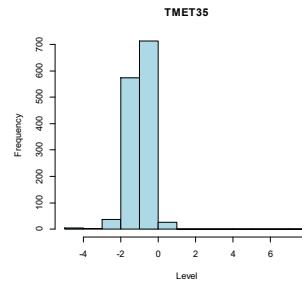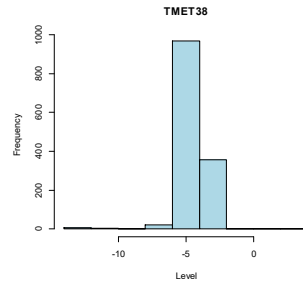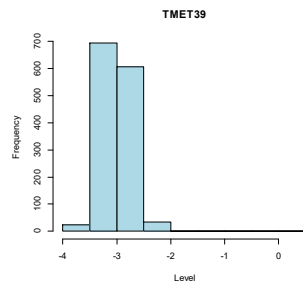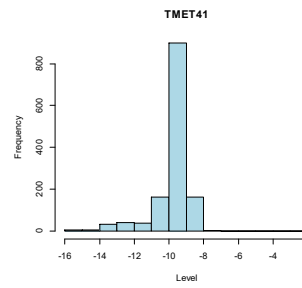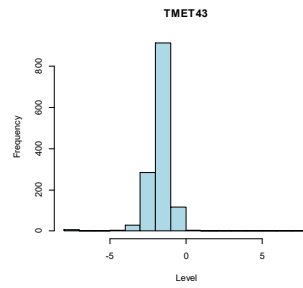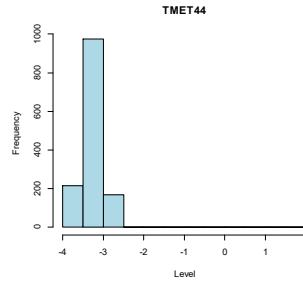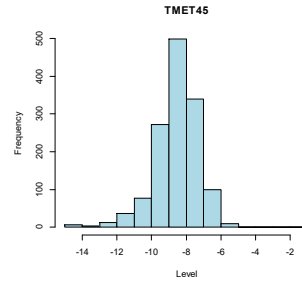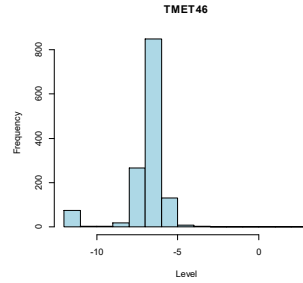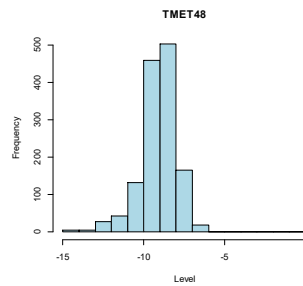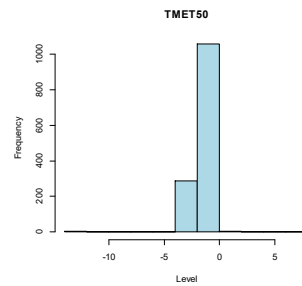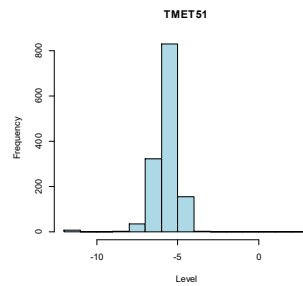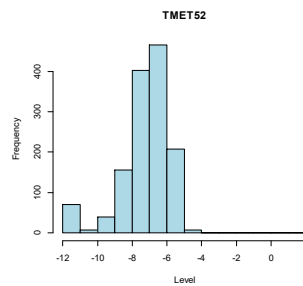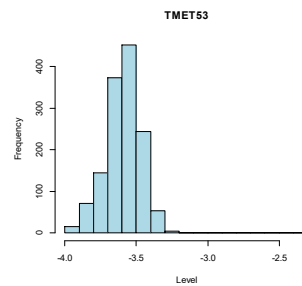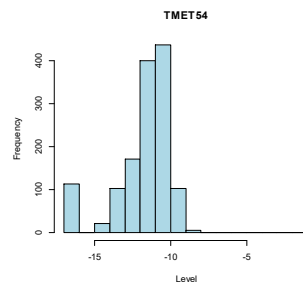

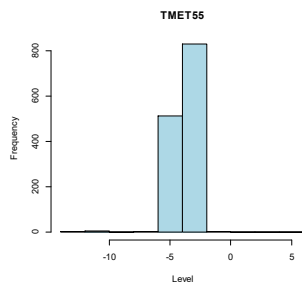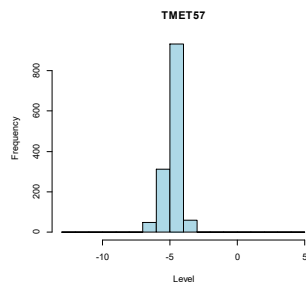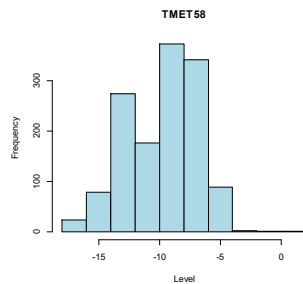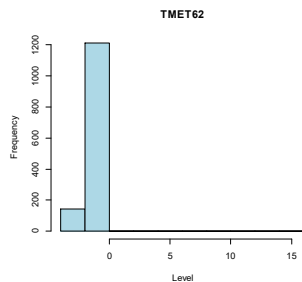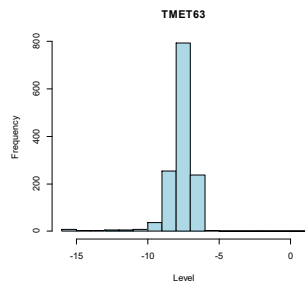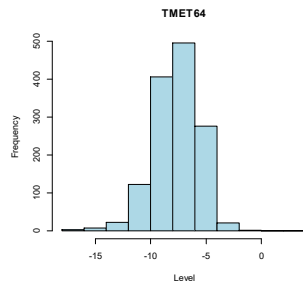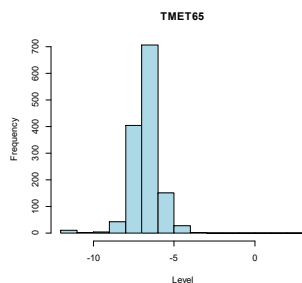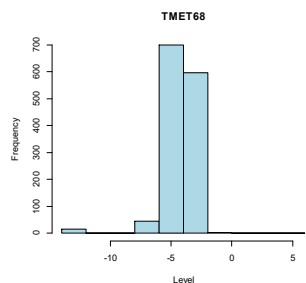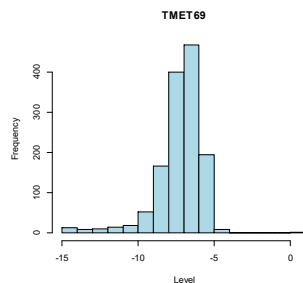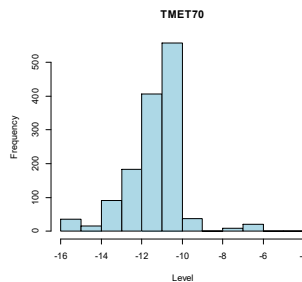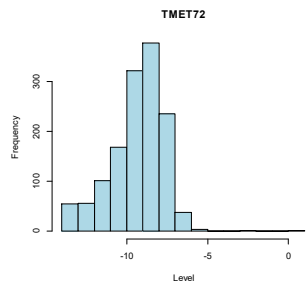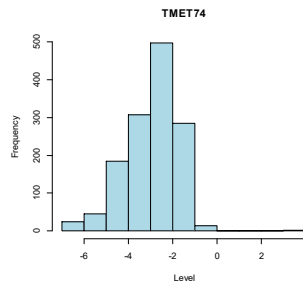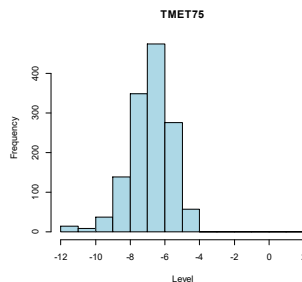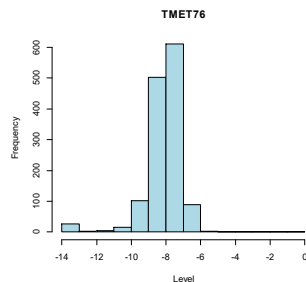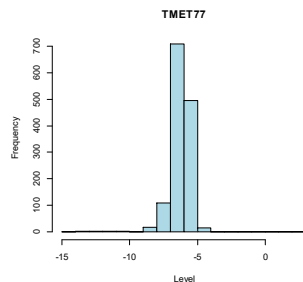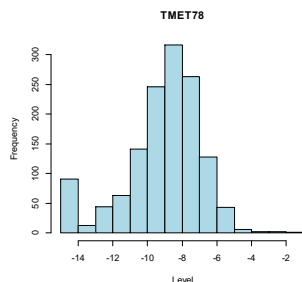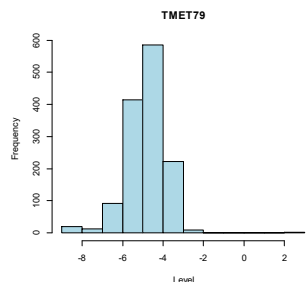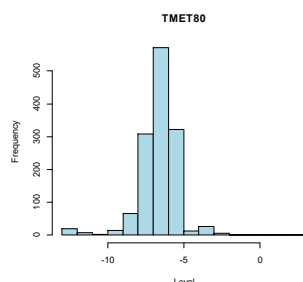

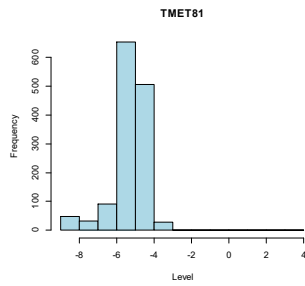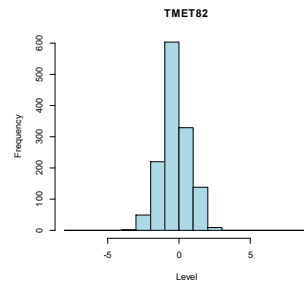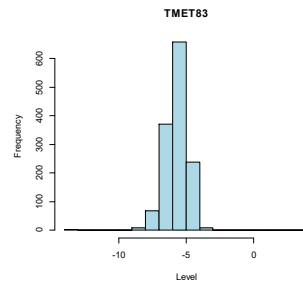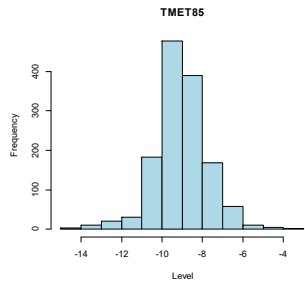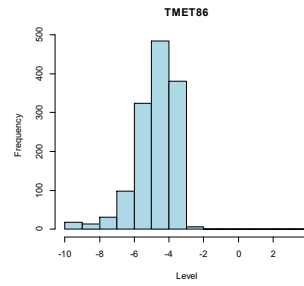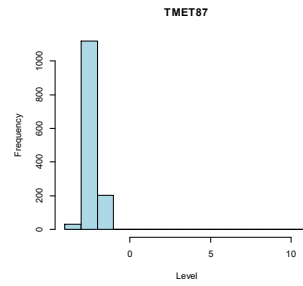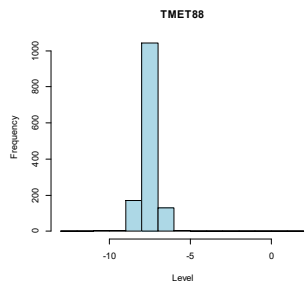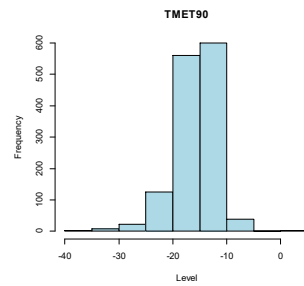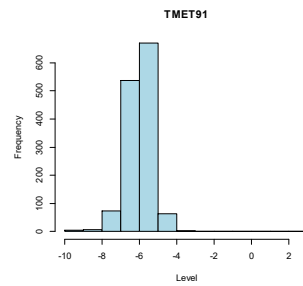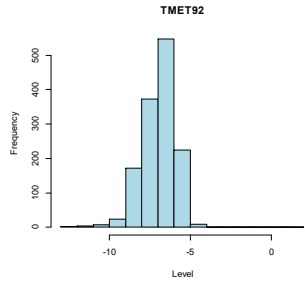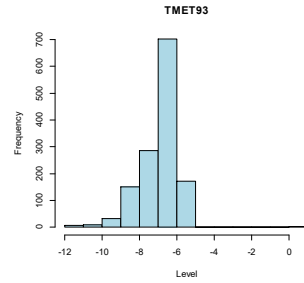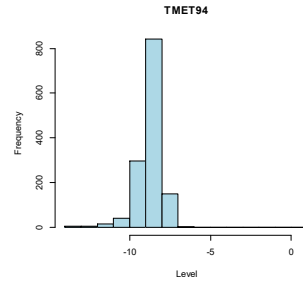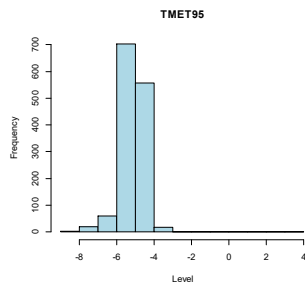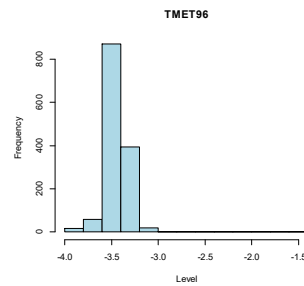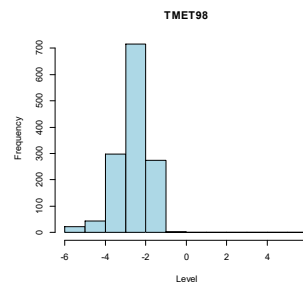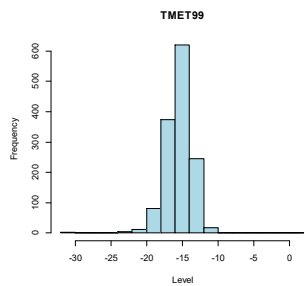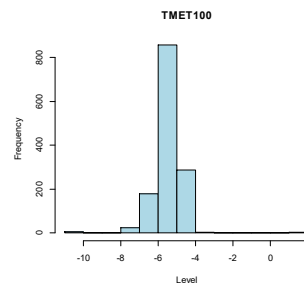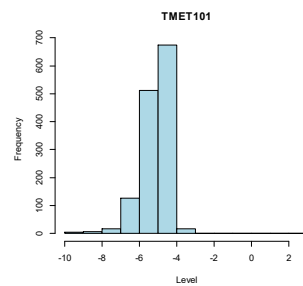

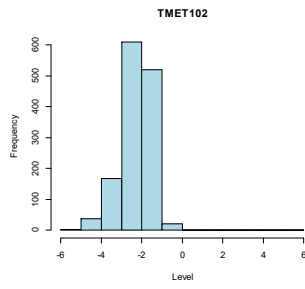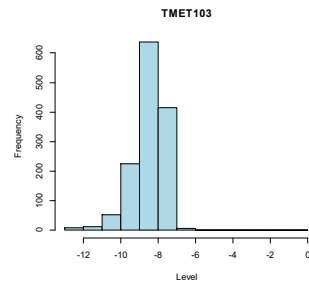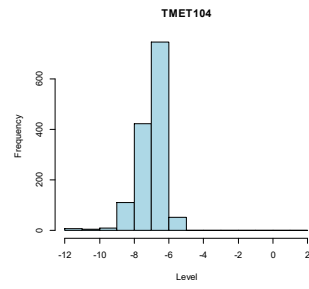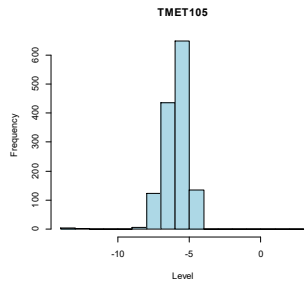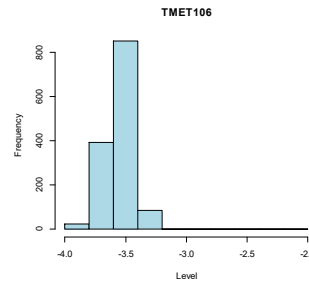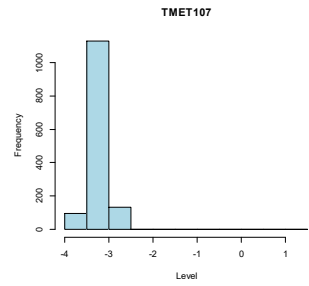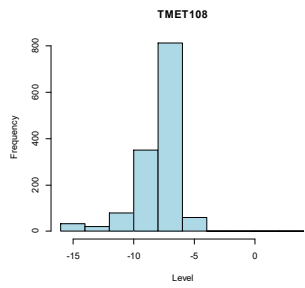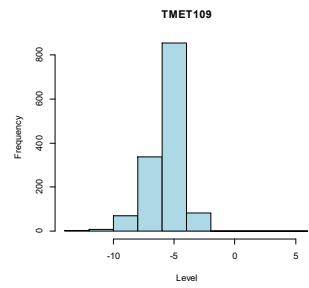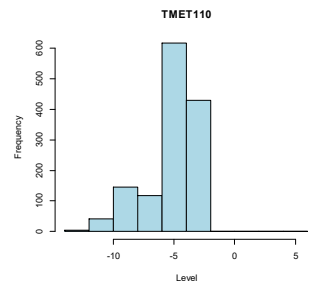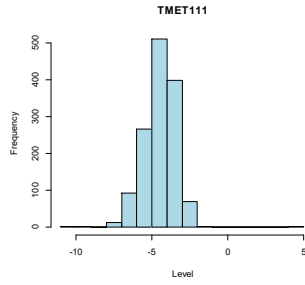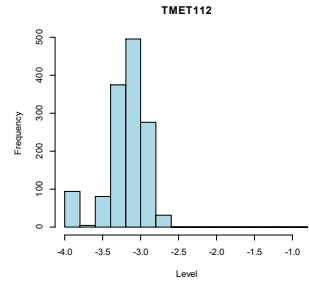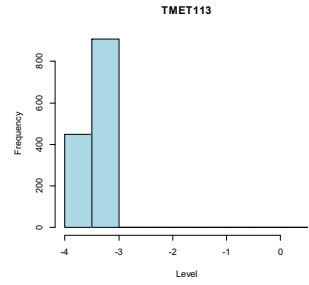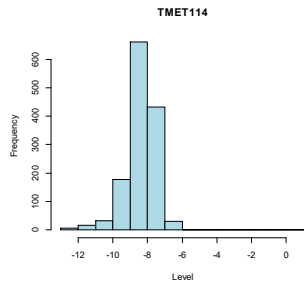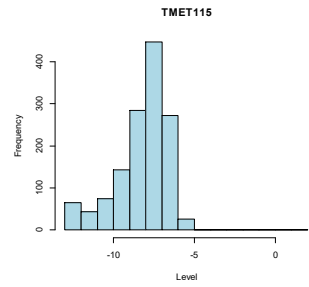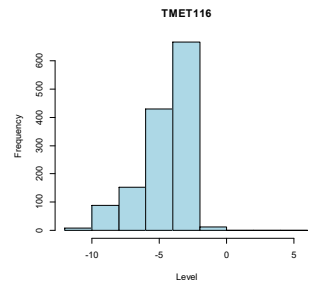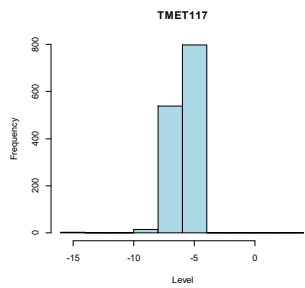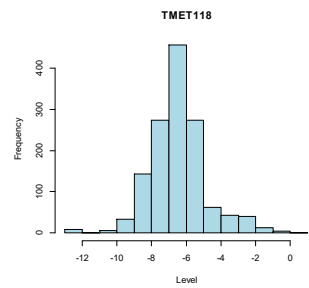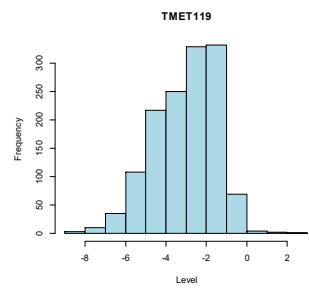

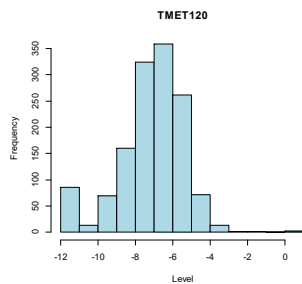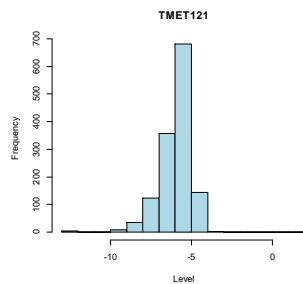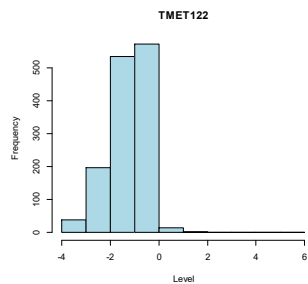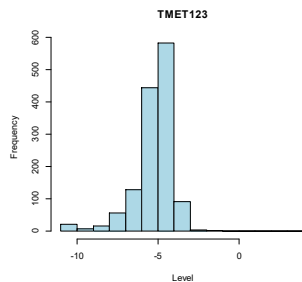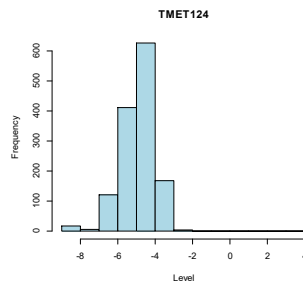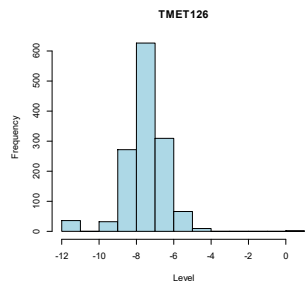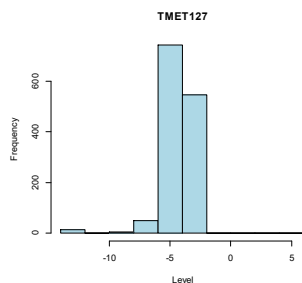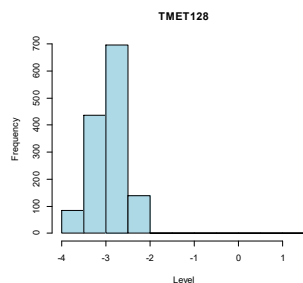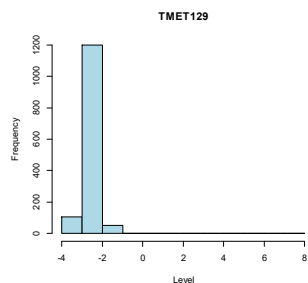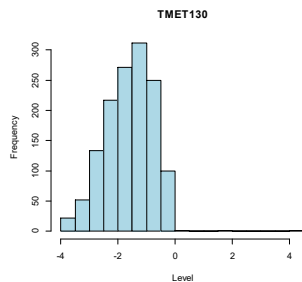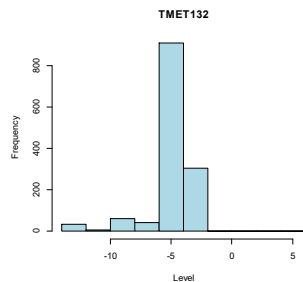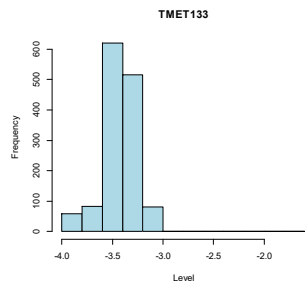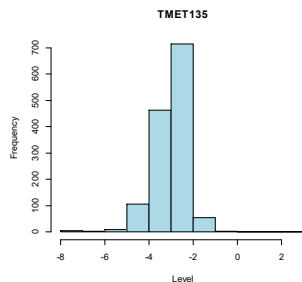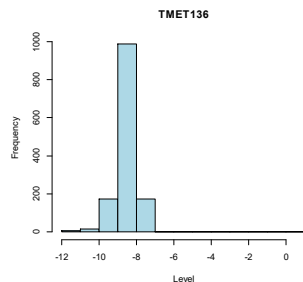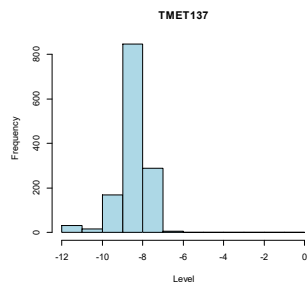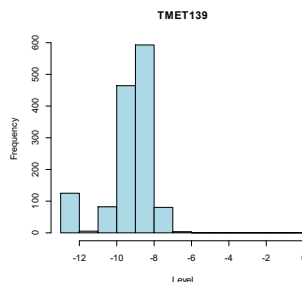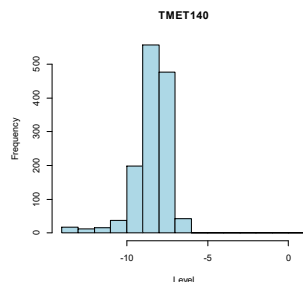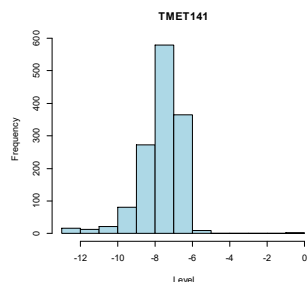

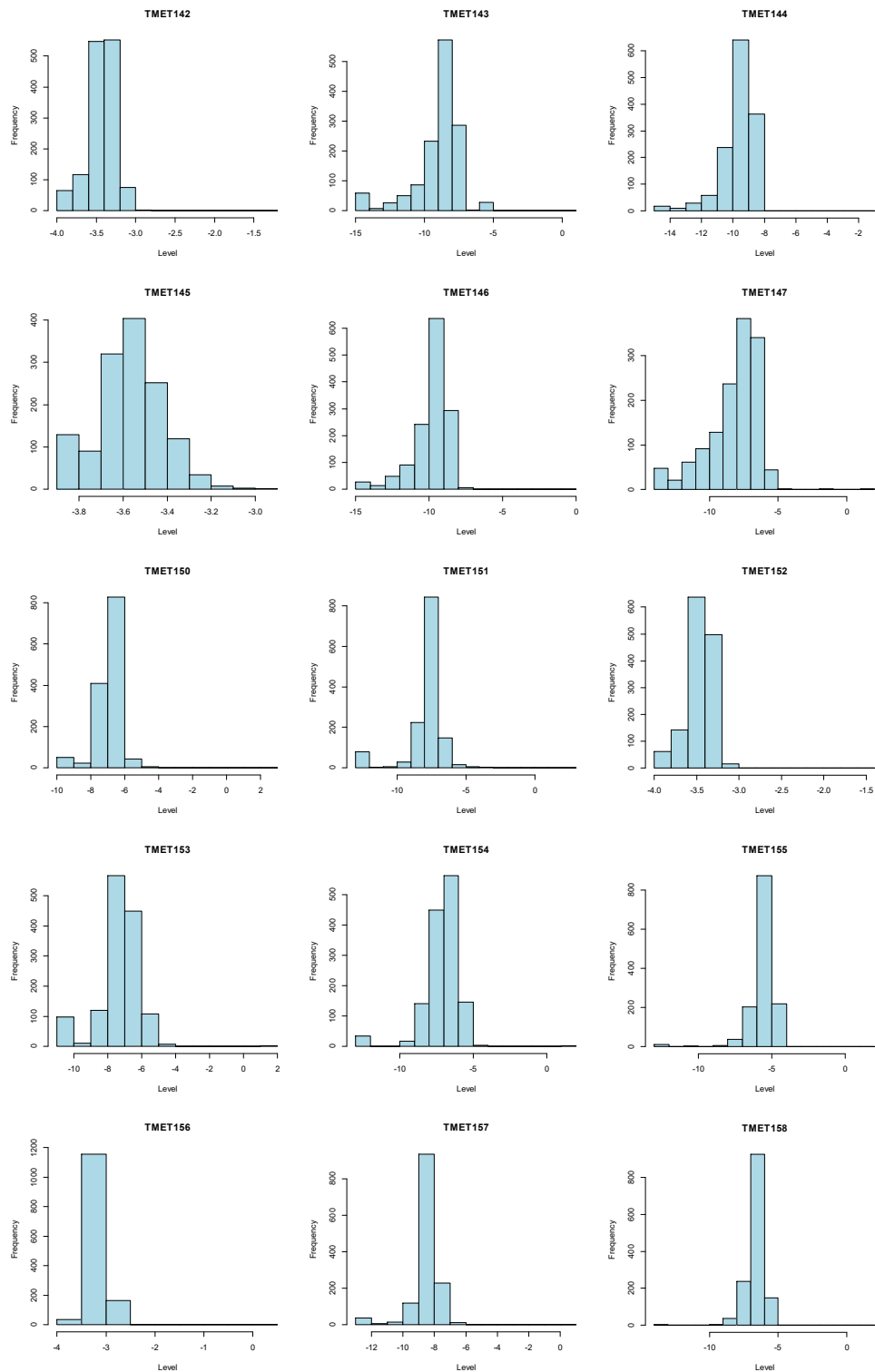

**S1 Fig.** Histograms of metabolites (after box-cox transformation) from 1<sup>st</sup> sampling date.

Supplement: S1 Fig — (PDF) [file pone.0246510.s001.pdf]

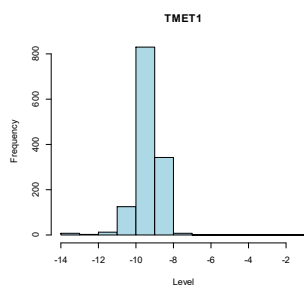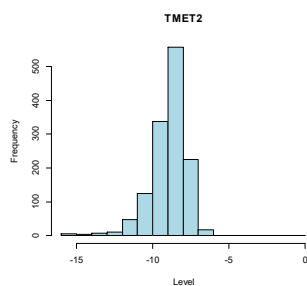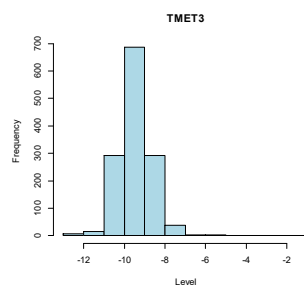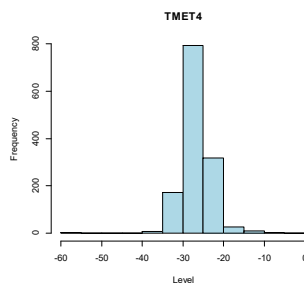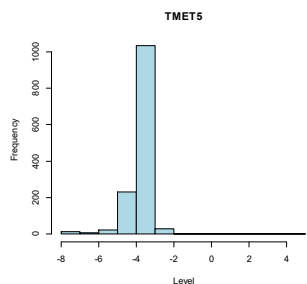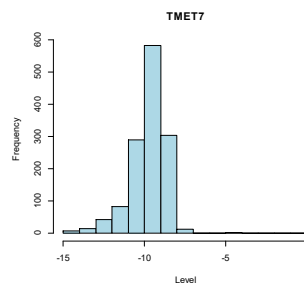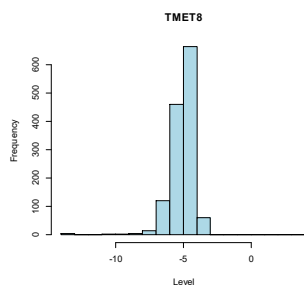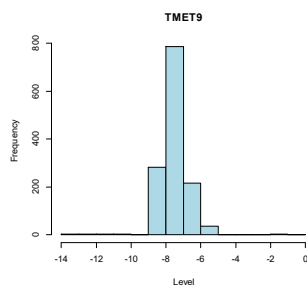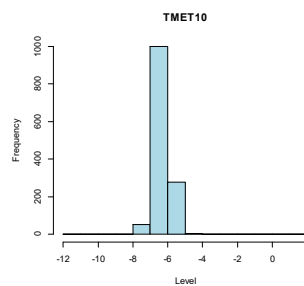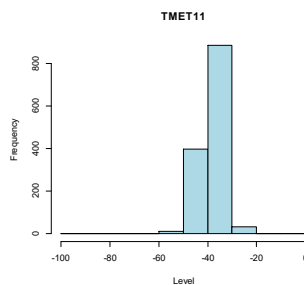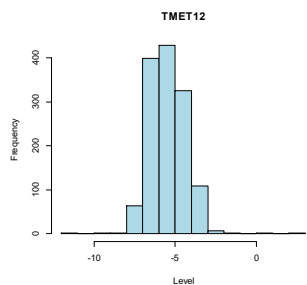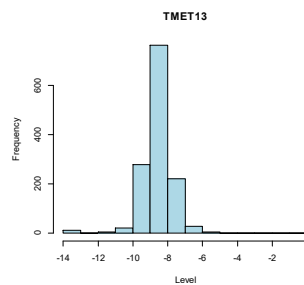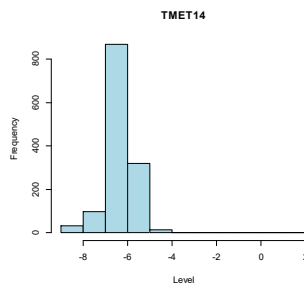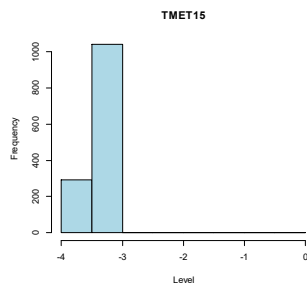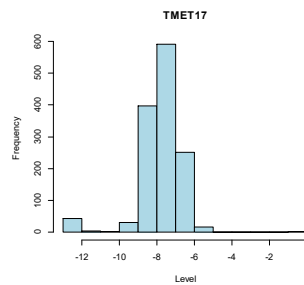

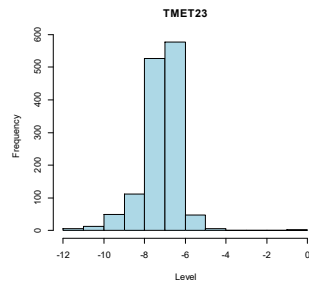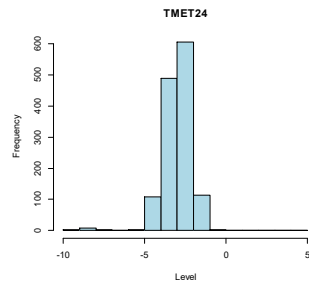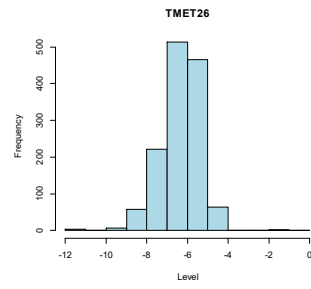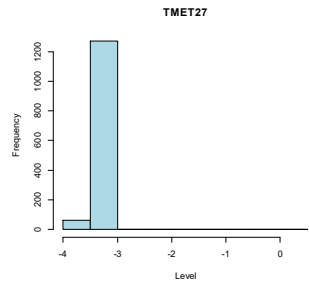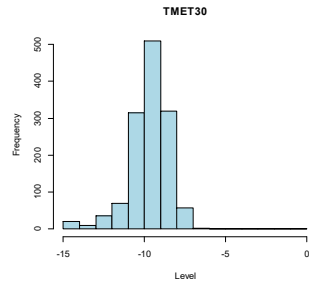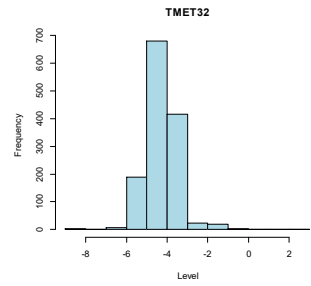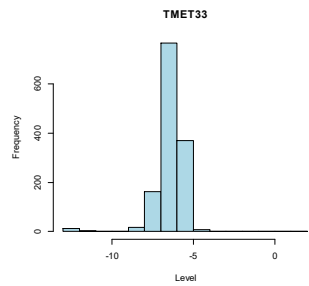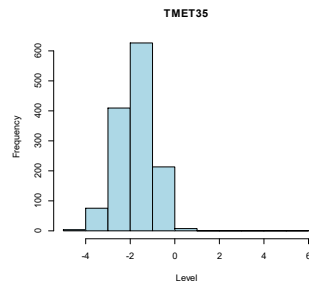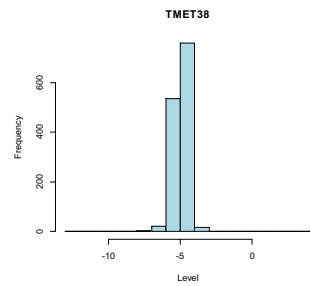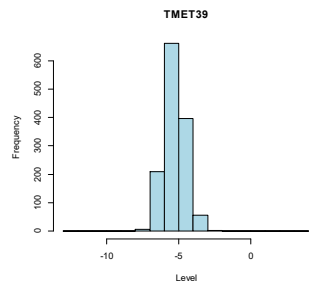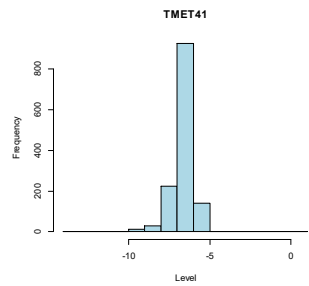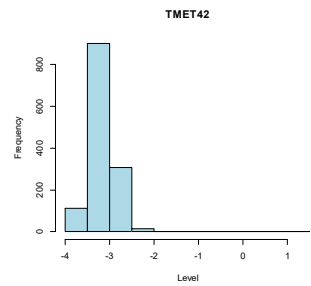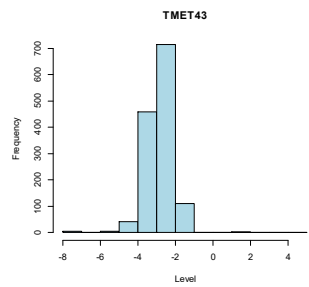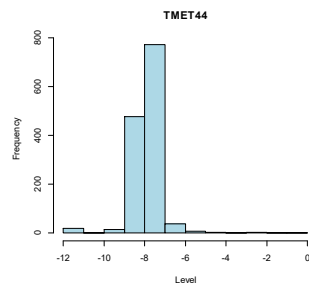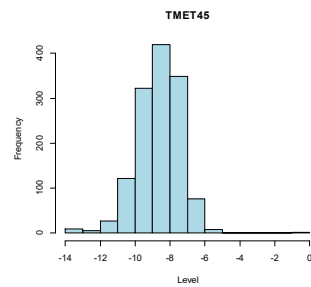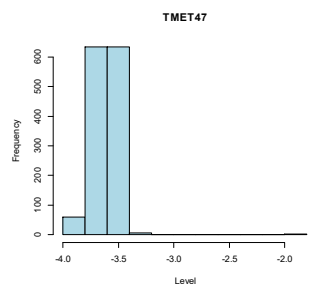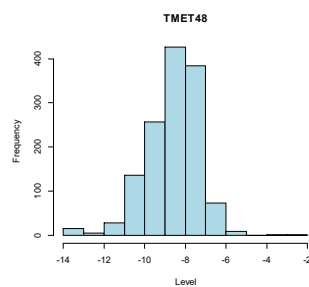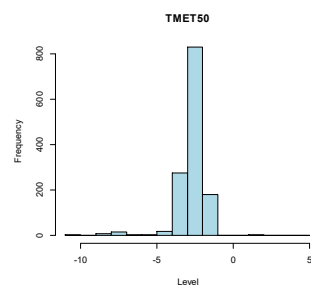

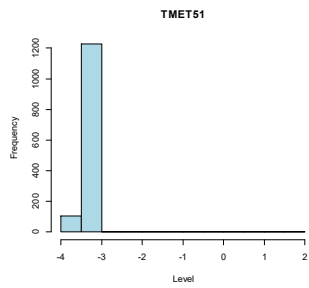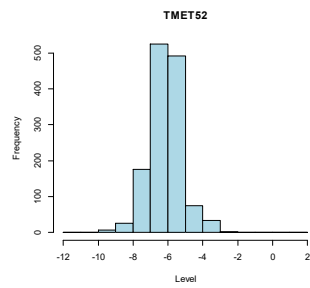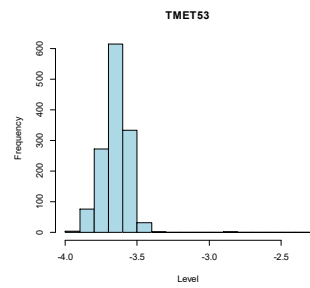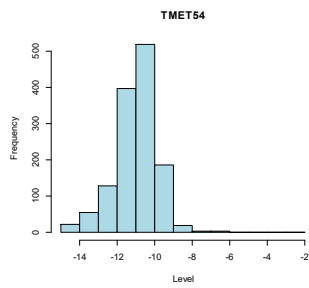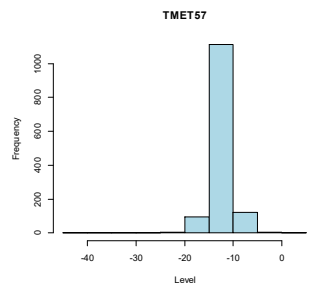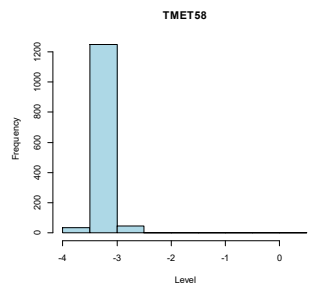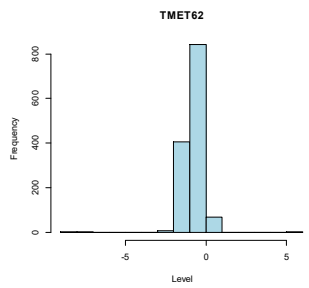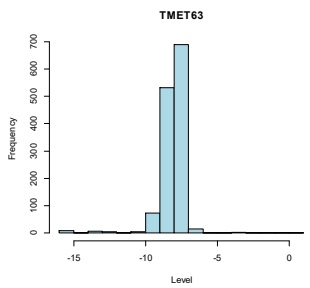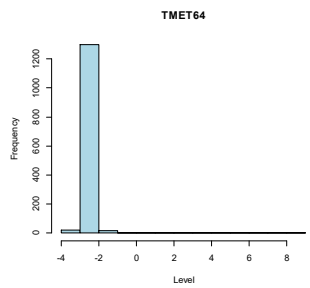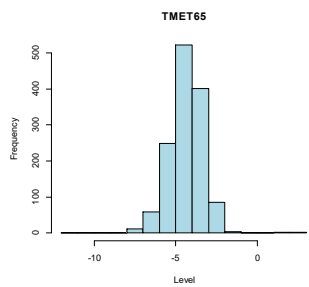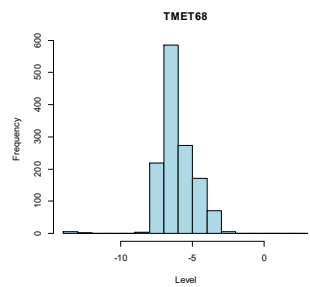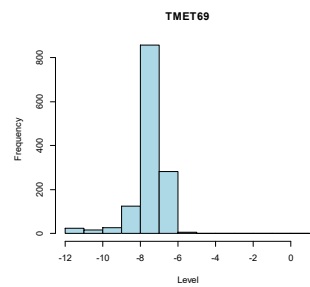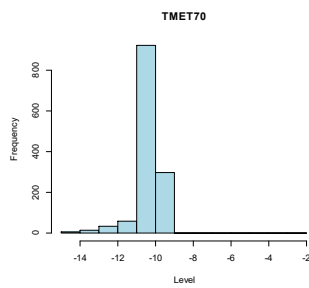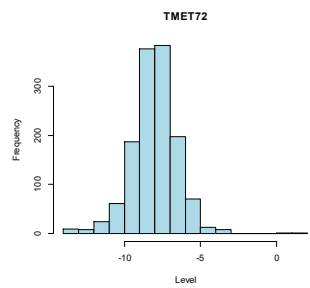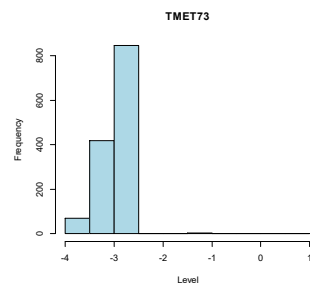

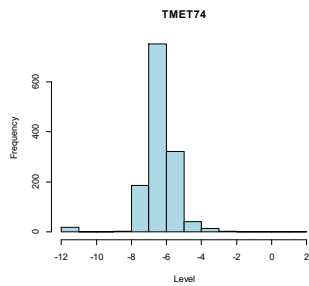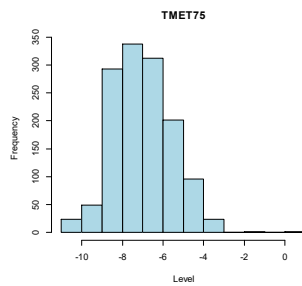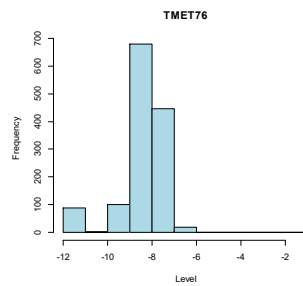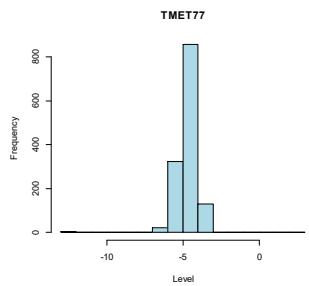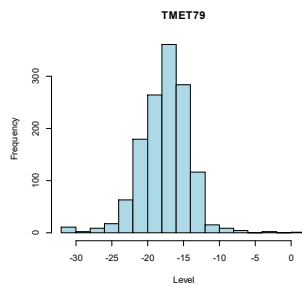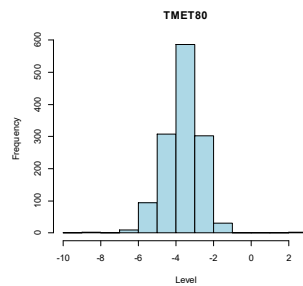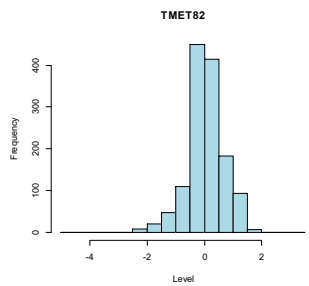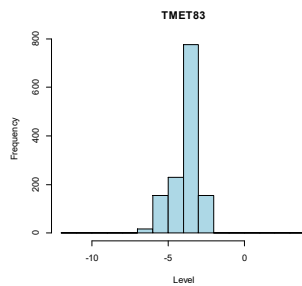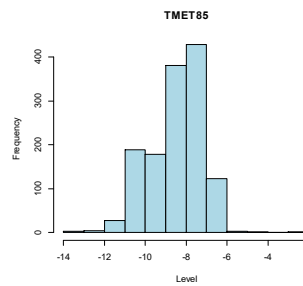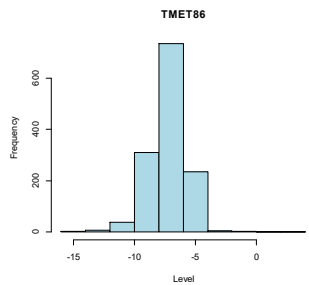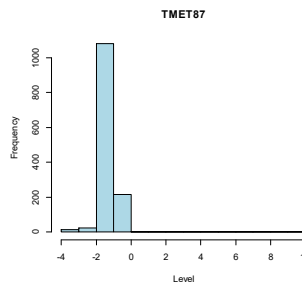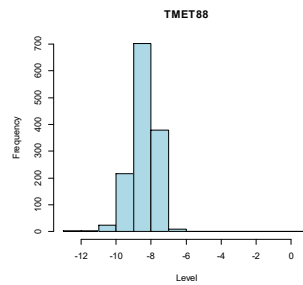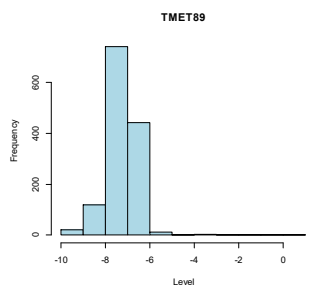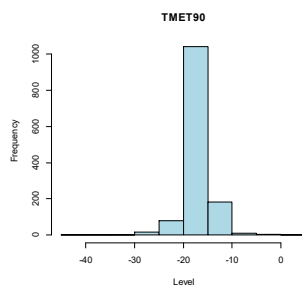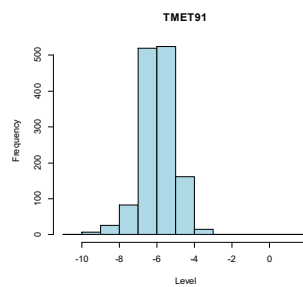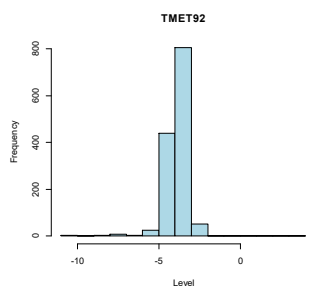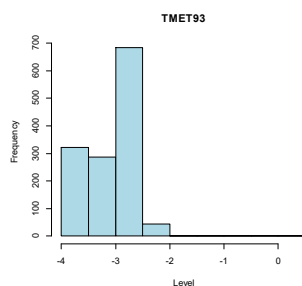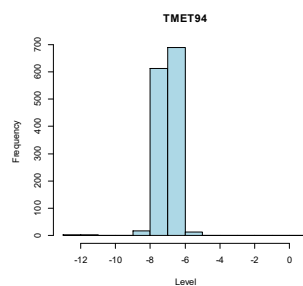

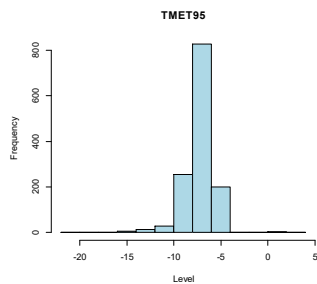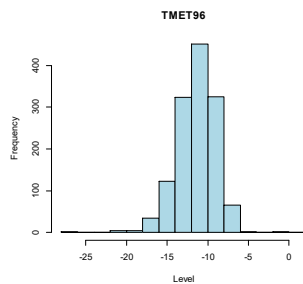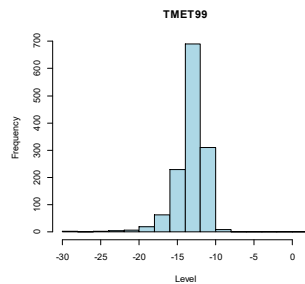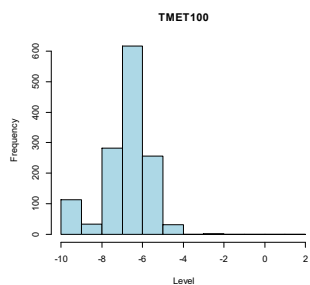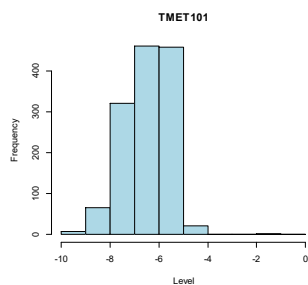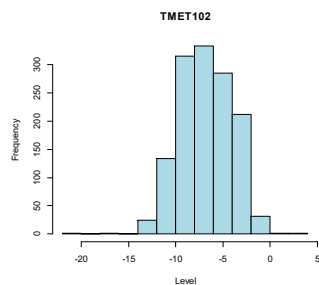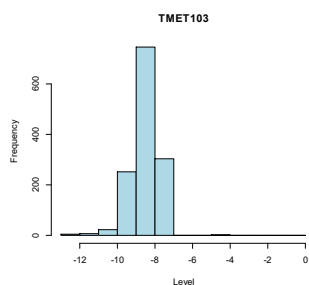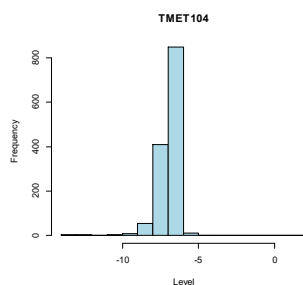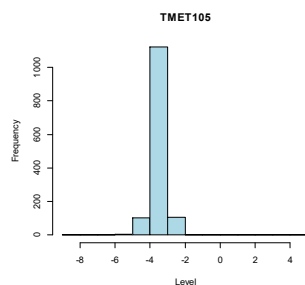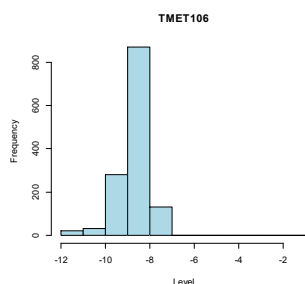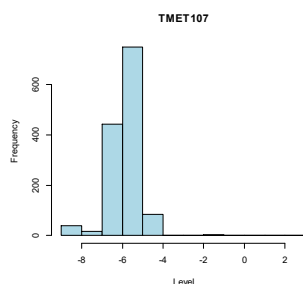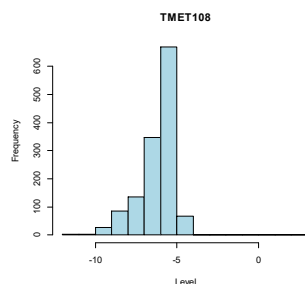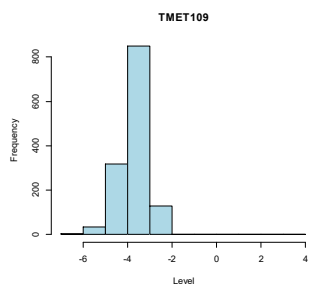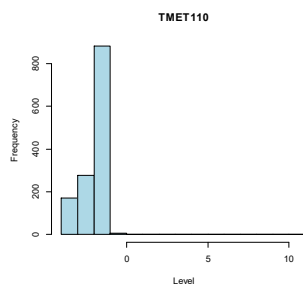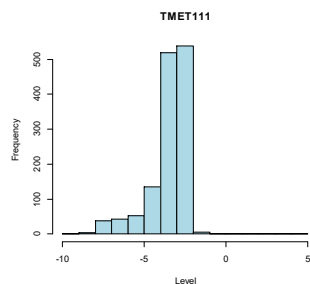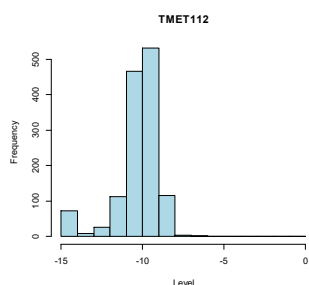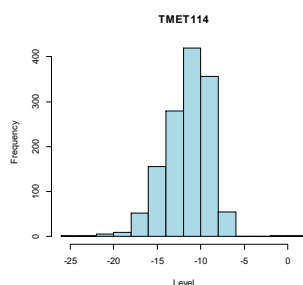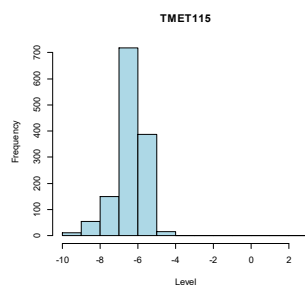

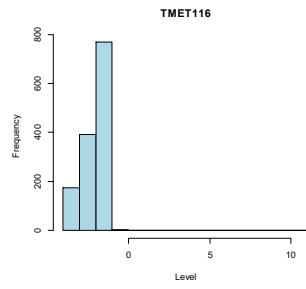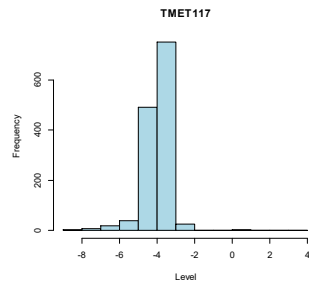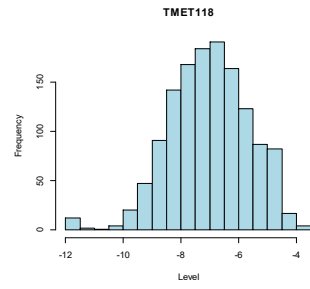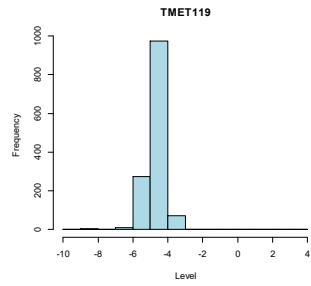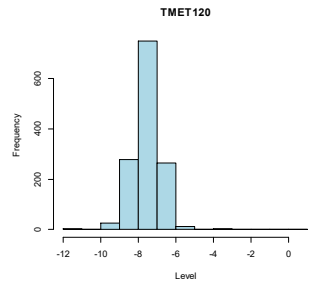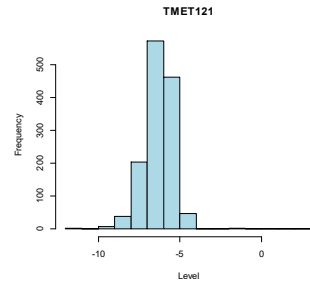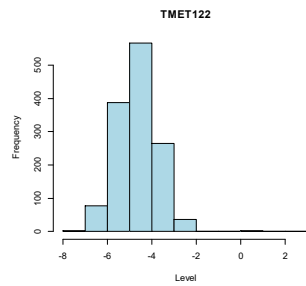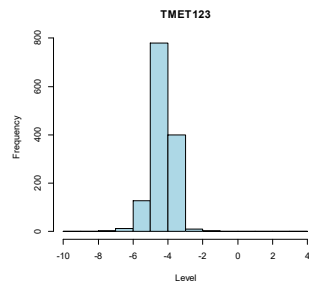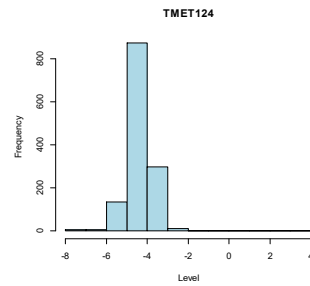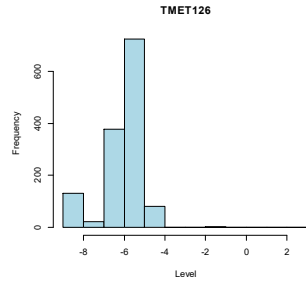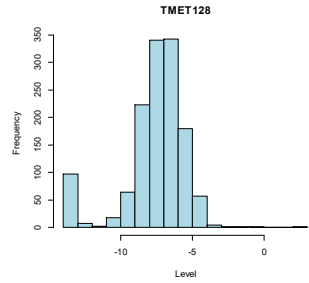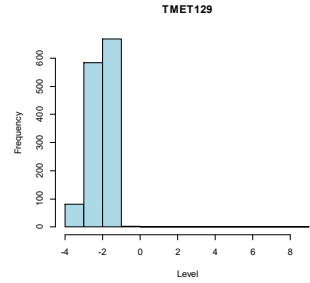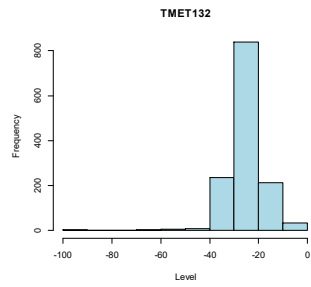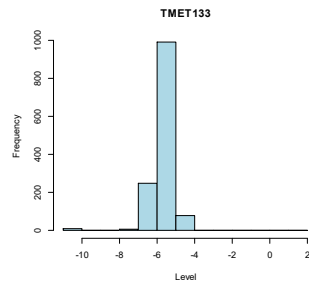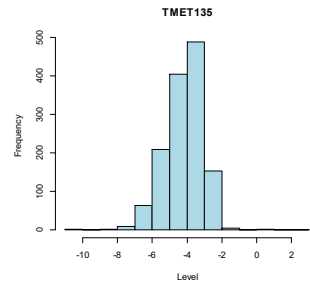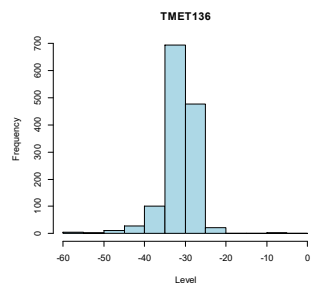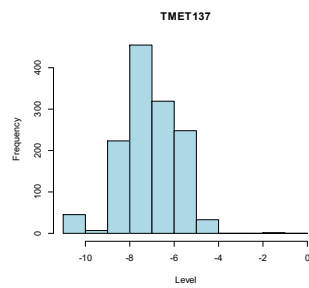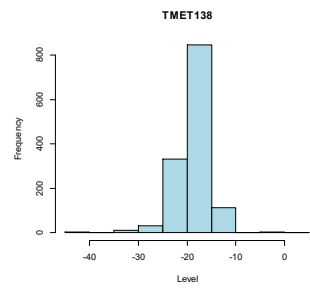

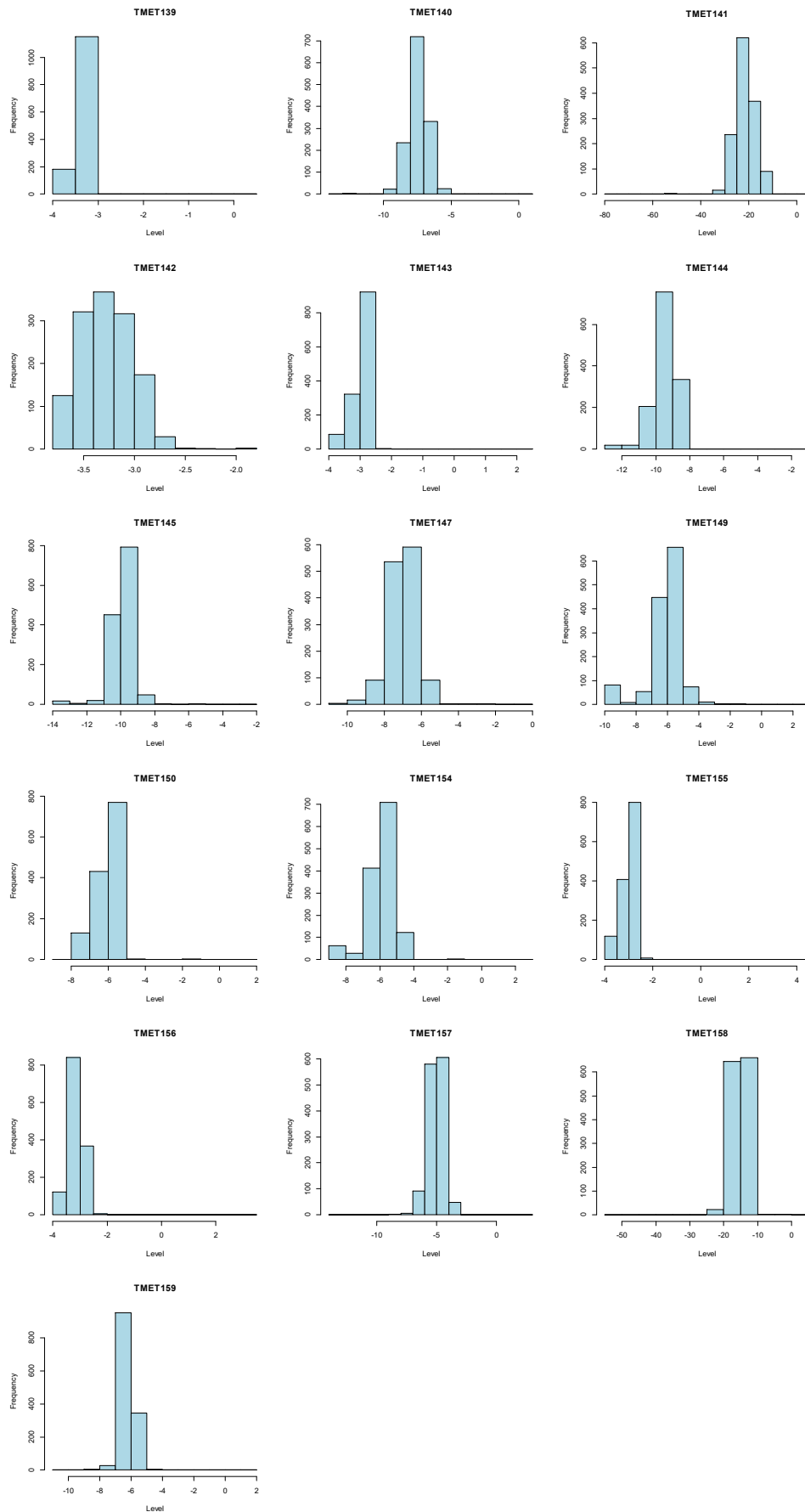

**S2 Fig.** Histograms of metabolites (after box-cox transformation) from 2<sup>nd</sup> sampling date.

Supplement: S2 Fig — (PDF) [file pone.0246510.s002.pdf]
