# Supplementary material for: Genome-wide association study on metabolite accumulation in a wild barley NAM population reveals natural variation in sugar metabolism
Source: PLoS One. 2021 Feb 16;16(2):e0246510. doi: 10.1371/journal.pone.0246510 (PMC7886226; doi:10.1371/journal.pone.0246510)
Supplement: S3 Fig — (PDF) [file pone.0246510.s003.pdf]

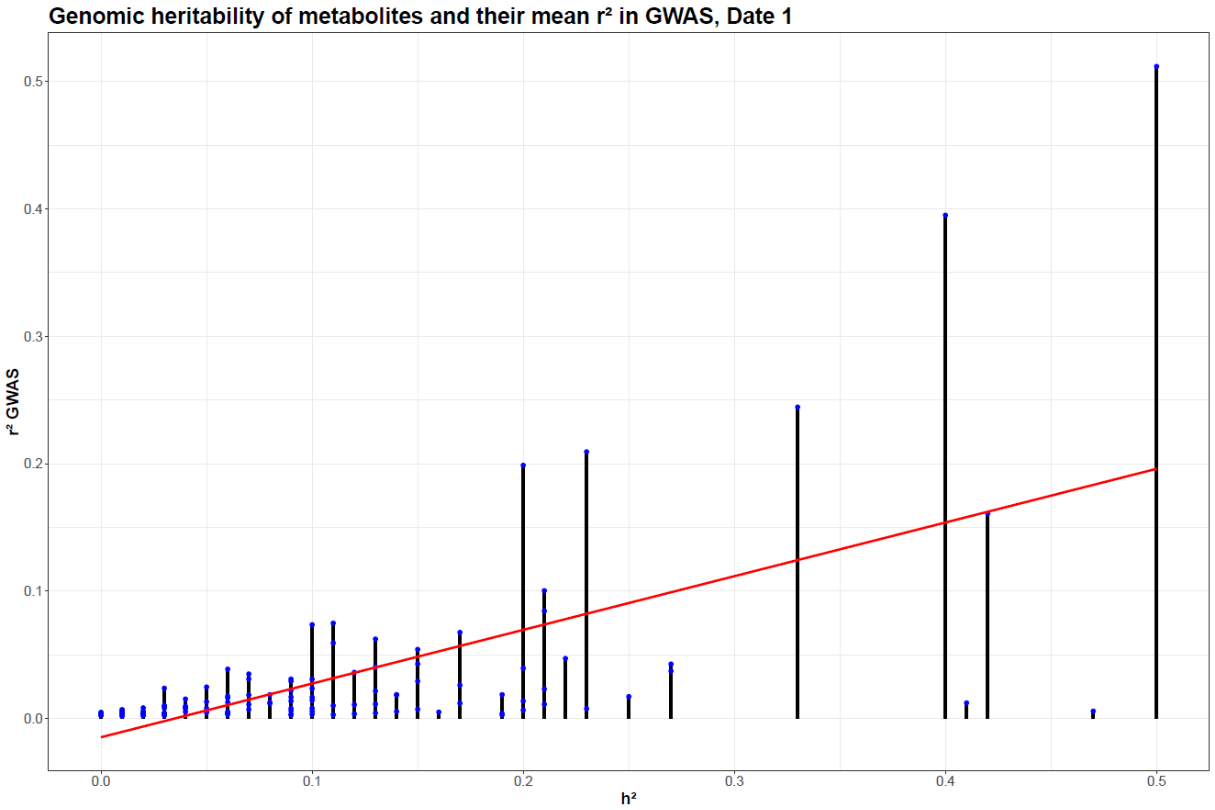

S3 Fig. Estimated mean r^2 value of metabolites in GWAS (r^2 GWAS) plotted against SNP based heritability (h^2) of metabolites, 1st sampling date. Regression line (red line) $r = 0.63$. Blue dots indicate each metabolite's r^2 GWAS on the y-axis and h^2 on the x axis.
