# Supplementary material for: Genome-wide association study on metabolite accumulation in a wild barley NAM population reveals natural variation in sugar metabolism
Source: PLoS One. 2021 Feb 16;16(2):e0246510. doi: 10.1371/journal.pone.0246510 (PMC7886226; doi:10.1371/journal.pone.0246510)
Supplement: S4 Fig — (PDF) [file pone.0246510.s004.pdf]

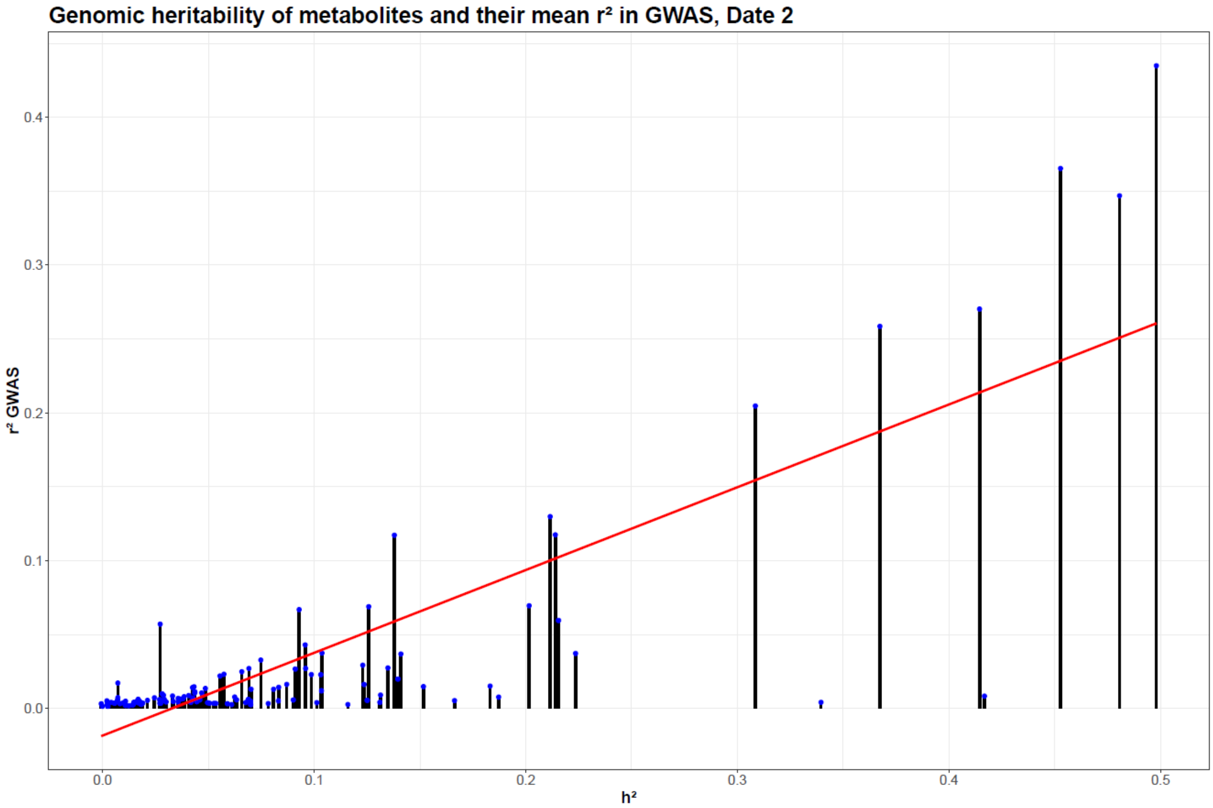


**S4 Fig.** Estimated mean $r^2$ value of metabolites in GWAS ($r^2$ GWAS) plotted against SNP based heritability ($h^2$) of metabolites, 2nd sampling date. Regression line (red line) r = 0.81. Blue dots indicate each metabolite's $r^2$ GWAS on the y-axis and $h^2$ on the x axis.
