# Supplementary material for: Genome-wide association study on metabolite accumulation in a wild barley NAM population reveals natural variation in sugar metabolism
Source: PLoS One. 2021 Feb 16;16(2):e0246510. doi: 10.1371/journal.pone.0246510 (PMC7886226; doi:10.1371/journal.pone.0246510)
Supplement: S6 Fig — (PDF) [file pone.0246510.s006.pdf]

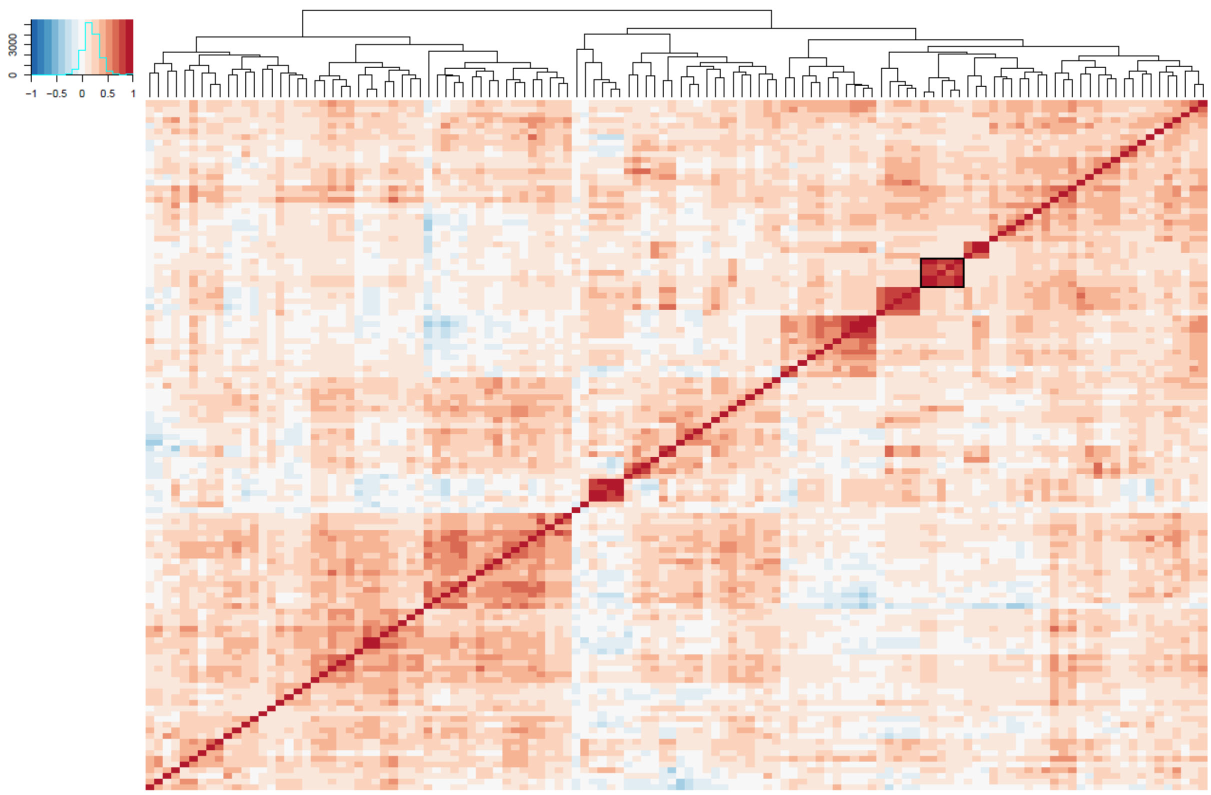

S6 Fig. Heatmap of correlation pattern among metabolites from 2nd sampling date. The black box indicates a correlation hotspot of the sugar and sugar-like metabolites, including these for which mQTLs were obtained in the present study.
